# Effectiveness of interventions for the management of multimorbidity in primary care and community settings: systematic review and meta-analysis

**DOI:** 10.1093/fampra/cmaf085

**Published:** 2025-11-11

**Authors:** Yen Wei Lim, Ibrahim S Al-Busaidi, Richelle Caya, Alessio Bricca, Dee Mangin, Ross Wilson, J Haxby Abbott

**Affiliations:** Department of Surgical Sciences, Centre for Musculoskeletal Outcomes Research, University of Otago, Dunedin 9054, New Zealand; Department of Primary Care and Clinical Simulation, University of Otago, Christchurch 8140, New Zealand; Department of Surgical Sciences, Centre for Musculoskeletal Outcomes Research, University of Otago, Dunedin 9054, New Zealand; Department of Sports Science and Clinical Biomechanics, Research Unit of Musculoskeletal Function and Physiotherapy, University of Southern Denmark, DK-5230 Odense M, Denmark; Department of Physiotherapy and Occupational Therapy, The Research and Implementation Unit PROgrez, Næstved-Slagelse-Ringsted Hospitals, 4200 Slagelse, Denmark; Department of Primary Care and Clinical Simulation, University of Otago, Christchurch 8140, New Zealand; Department of Surgical Sciences, Centre for Musculoskeletal Outcomes Research, University of Otago, Dunedin 9054, New Zealand; Department of Surgical Sciences, Centre for Musculoskeletal Outcomes Research, University of Otago, Dunedin 9054, New Zealand

**Keywords:** multimorbidity, systematic review, meta-analysis, primary health care, patient care management, treatment outcomes

## Abstract

**Background:**

Multimorbidity—the co-existence of two or more chronic health conditions in the same individual, without reference to an index condition—has become a global health issue and creates enormous pressure on the healthcare system. This review aimed to summarize evidence on the effectiveness of interventions used to manage people with multimorbidity.

**Methods:**

MEDLINE, EMBASE, CINAHL, Cochrane Library, two trials registers, and grey literature were searched for studies of adults with multimorbidity receiving care in primary or community care settings up to 30 September 2024. Two reviewers independently screened studies for eligibility, extracted data, and assessed risk of bias and study certainty. Interventions were categorized as medicines management (MM), support for self-management (SSM), or care coordination plus support for self-management (CC + SSM). Meta-analyses for primary outcomes (health-related quality of life, healthcare utilization, and healthcare costs) were conducted.

**Results:**

From 10 272 titles screened, 33 eligible studies (this review: 18, previous review: 15; MM: 6, SSM: 9, CC + SSM: 18) were identified, of which 26 studies with 9449 participants were included in meta-analysis. Overall, there was little significant evidence of benefit of the interventions compared with usual care for most outcomes. SSM was associated with lower hospitalization risk and medication costs, but slightly more emergency department (ED) visits; and CC + SSM with better SF-12 PCS score, lower hospitalization risk and fewer ED visits, but more outpatient and general practitioner visits.

**Conclusion:**

This review found some suggestions of improved outcomes and reduced healthcare utilization (especially hospitalization) for these interventions. There is a paucity of evidence reporting on health outcomes, especially healthcare costs, in the management of multimorbidity.

Key messages

- This systematic review offers updated evidence on the effectiveness of interventions for multimorbidity in primary care.
- Our findings reveal little evidence of improved outcomes related to healthcare utilization.
- We found evidence gaps in multimorbidity research, especially around the cost of interventions for the management of multimorbidity.

## Background

Multimorbidity—the co-existence of two or more chronic health conditions in the same individual [1, 2]—has become a growing public health concern [3]. The global prevalence of multimorbidity in community settings is 33% [3, 4], and this rate is increasing due to an aging population [5]. While multimorbidity is most common in the older population—half of those aged 65 years and older has multimorbidity [6]—people under 65 years old also experience a substantial multimorbidity burden and the absolute number of people with multimorbidity in this group is greater than those aged 65 years and older [7]. Multimorbidity may occur at an earlier age especially in patients with high socioeconomic deprivation or lower socioeconomic status, who are often vulnerable people, having low health literacy and may not have equitable healthcare access [8, 9]. For patients, multimorbidity is associated with reduced quality of life, increased morbidity and mortality, and severe financial burden on patients and their families [1, 9, 10]. Over half (58%) of people attending primary care have multimorbidity and over three quarters (78%) of consultation are with patients living with multimorbidity [11]. Thus, multimorbidity creates substantial additional pressure on the healthcare system as more, and more complex, care services are needed [1, 9].

Care for multimorbidity is patient-centred and family-centred and does not focus to any single index condition [10]. Instead, it emphasizes the need to focus on the biopsychosocial issues that usually accompany multimorbidity. Interventions for multimorbidity need to have a generic focus that will work across a broad range of conditions [2, 12]. A related term, comorbidity, is used when there is a defined index condition with other linked conditions; clinical care for comorbidity is usually focused on the index condition [13, 14].

A previous systematic review by Smith *et al.* [2] of interventions for improving health outcomes in patients with multimorbidity identified 16 randomized trials with a total of 4753 participants published up to September 2019 [2]. Results of interventions were disappointing: the review found little or no difference on the main outcomes of health-related quality of life (HRQoL) and mental health outcomes or on secondary outcomes such as healthcare utilization and medicine outcomes. A small number of included studies reported that care coordination may improve patient experience of care and self-management support may improve patient health behaviours, but there remain substantial uncertainties [2].

Multimorbidity research is a rapidly growing field; substantial new evidence has been published since 2019 that might provide better indications of approaches that improve outcomes and reduce the other uncertainties identified by Smith *et al.* Furthermore, little is known about the effects of these interventions on healthcare utilization or costs. This systematic review aimed to address the existing evidence gap by providing an up-to-date synthesis of both the clinical and economic effectiveness of interventions used to improve health outcomes for people with multimorbidity in primary care and community settings. Whereas the previous review focused on health-related quality of life (HRQoL) as the primary outcome, with healthcare utilization and healthcare costs among the secondary outcomes, this update considers these as primary outcomes alongside HRQoL. This information is necessary to inform policy and funding decisions on affordable and efficient management of the growing burden of multimorbidity.

## Methods

### Registration and reporting

In this study we updated the systematic review of Smith *et al.* to include studies published up to September 2024. Methods for this study were identical to those of the earlier review, except for the additional inclusion of healthcare utilization and healthcare costs as primary outcomes alongside HRQoL. This update was guided by the recommendations in the *Cochrane Handbook for Systematic Reviews of Interventions* [15] and reported according to the Preferred Reporting Items for Systematic Review and Meta-analysis (PRISMA) checklist [16]. The review is registered with the International Prospective Register of Systematic Reviews (PROSPERO) database [CDR42023476278]. The methods of this review have been reported in detail in our protocol published in the Open Science Framework [17].

### Search strategy

We searched published articles on MEDLINE (Ovid), EMBASE (Ovid) and CINAHL (EBSCO) online databases, registered trials and systematic reviews in the Cochrane Library, and two trials registers (ClinicalTrials.gov and WHO International Clinical Trials Registry Platform), covering from 1 January 2019 up to 30 September 2024. We also searched grey literature via the Open Grey database, the National Library of Medicine, and the International Research Community on Multimorbidity. Additional references were located through searching the reference lists of articles identified and searching of relevant guidelines was conducted to ensure any additional relevant data were not missed. Search strategies are available in Appendix S1.

### Selection criteria

Our systematic review included studies reported as full-text papers and published in English. Study designs eligible for inclusion were randomized trials, non-randomized trials, controlled before-after studies, and interrupted time series analyses, meeting Cochrane Effective Practice and Organization of Care (EPOC) quality criteria [18]. Studies of adults with multimorbidity, defined as co-existence of two or more chronic conditions (health problems that require ongoing management over a period of years or decades [19]) in the same individual receiving care in a primary or community care setting were included. Chronic conditions can be physical non-communicable disease (such as cardiovascular disease, diabetes and cancer), infectious diseases of long duration (such as HIV), and mental health conditions (such as depression) [1]. Any type of intervention based in primary care and community settings that was specifically directed towards a group of people defined as having multimorbidity was included. Rather than solely targeting index conditions, interventions for multimorbidity should adopt a more generic focus that is applicable across a broad range of conditions [2]. Such an approach is critical for the development and evaluation of effective interventions and for ensuring their generalizability [20]. Interventions identified in the review would be complex and multifaceted, and were categorized into three broad groups: medicines management (MM), self-management support (SSM), and care-coordination plus self-management support (CC + SSM).

We excluded studies in which inclusion was based on comorbidity with a specific index condition. Studies in which multimorbidity was assumed to be the norm because of individuals' age and where interventions did not target multimorbidity specifically were excluded. In addition, professional educational interventions or research initiatives with no specified structured clinical care delivered to an identified group of people with multimorbidity were excluded.

### Review processes

After deduplication, articles were screened for eligibility using JBI SUMARI (System for the Unified Management of the Assessment and Review of Information). Two reviewers [YWL (all studies) and ISA or RC (half each)] screened the articles independently to identify studies meeting the predefined inclusion and exclusion criteria. The reviewers examined successively the titles, abstracts, and full texts of all possibly relevant articles identified by our searches. Excluded studies and reasons for exclusion of full texts were listed. Any disagreements were resolved through discussion and consensus.

### Data extraction, risk of bias assessment, and quality assessment

A data extraction form created by the authors was used by two independent reviewers (YWL and ISA or RC) to extract information required for the review. In addition to the articles identified in our search, YWL also extracted data (checked by ISA) from the articles included in Smith *et al*. The reviewers recorded data on study characteristics (the name of the first authors, country of publication, setting of the study, number of patients allocated to the intervention and comparator groups respectively, duration of the intervention or follow-up, study design, multimorbidity operational definition, and funding sources), participant characteristics (age, percentage of gender, number of conditions/diseases), intervention and comparator characteristics using the Template for Intervention Description and Replication (TIDieR) checklist [21], and outcome measurements. We grouped interventions into the same three categories as Smith *et al.*: MM, SSM, and CC + SSM. After data extraction was completed by each of the reviewers, the data were compiled. The reviewers discussed the inputs and revised the extracted data as necessary. Unresolved issues were resolved by involving a third reviewer (RC or ISA).

The revised Cochrane Risk-of-Bias Tool (RoB 2) was used to determine the risk of bias of all the included randomized controlled trials [22]. For cluster-randomized trials, the revised Cochrane Risk-of-Bias tool for Cluster-Randomized Trials (RoB 2 CRT) was used [23]. The Risk-of-Bias In Non-randomized Studies—of Interventions (ROBINS-I) tool was applied to assess the risk of bias for all non-randomized trials [24]. All risk of bias assessments were performed by two reviewers (YWL and ISA or RC) independently. Discrepancy check was carried out after the completion of risk of bias assessment by all the reviewers. Any disagreement between reviewers was resolved through discussion, or by a third reviewer (RC or ISA) if consensus could not be reached. Two reviewers (YWL and ISA) independently assessed the certainty of evidence for each outcome in accordance with the types of intervention using the Grading of Recommendations Assessment, Development and Evaluation (GRADE) criteria [25].

### Outcomes

The primary outcomes identified in this review were patients' HRQoL, healthcare utilization, and healthcare cost. HRQoL outcomes were categorized as health utility values (e.g. EQ-5D, SF-6D), SF-12 Physical Component Summary (PCS) and Mental Component Summary (MCS) scores, and other HRQoL measures. Healthcare utilization outcomes included any reported measures of healthcare utilization in the identified studies, such as number of hospitalizations, days spent in hospital, and number of emergency department visits. Healthcare cost outcomes included total healthcare costs, as well as the subcategory costs of specific healthcare services (such as cost of emergency department visits). Additional outcomes reported in the original review were included as secondary outcomes. These include health behaviours, medicines outcomes, psychosocial outcomes, clinical outcomes, adverse events, and clinicians' behaviours [2].

### Statistical analysis

Meta-analyses were undertaken when studies' participant populations, interventions, and outcomes were deemed to be compatible. Meta-analyses were performed using the *metafor* package [26] in the R statistical software (version 4.4.0) [27] for the primary outcomes using a random-effects model with generic inverse variance (no meta-analysis was planned for secondary outcomes). We performed meta-analyses for continuous outcomes using the mean difference (MD; when all studies reported the same outcome measure) or standardized mean difference (SMD; when studies used different outcome measures to assess the same domain), and for dichotomous outcomes using the odds ratio (OR).

Healthcare use and costs outcomes were standardized to values per year of follow-up to account for varying follow-up periods between studies. Missing data on standard deviations needed for meta-analysis were imputed using the best estimates of standard deviations from the included studies in this review. The *I*^2^ statistic was used to quantify the proportion of total variability due to between-study heterogeneity across the included studies. We pooled together in meta-analysis the longest follow-up available after randomization, for each outcome domain, as multimorbidity is a chronic condition requiring effective long-term management.

Sensitivity analyses were conducted by removing studies with high risk of bias for the outcomes assessed and removing non-randomized studies of interventions (NRSI).

## Results

### Literature search

**Figure 1.**
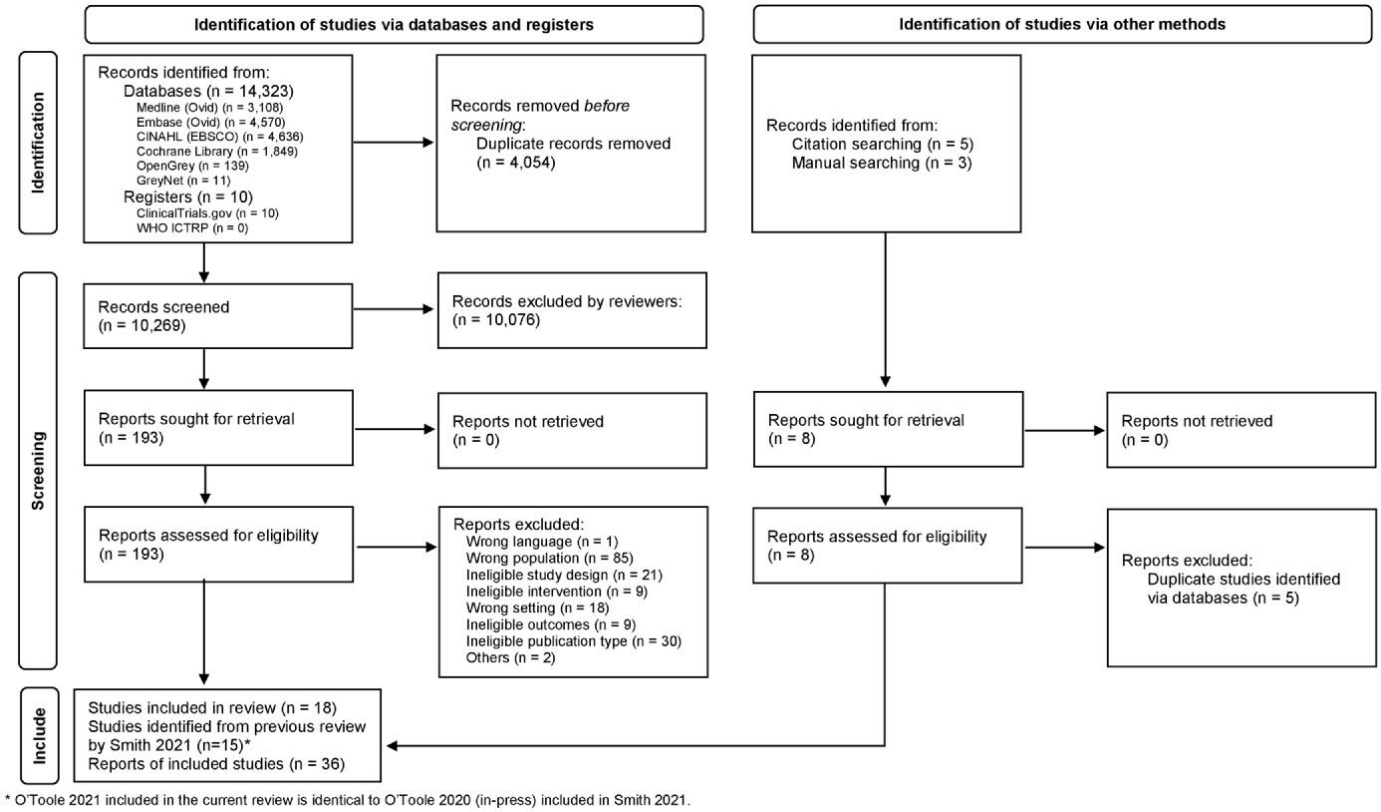
PRISMA flow diagram for study selection.

We identified 14 323 articles from our initial database search, 10 registered studies with full outcomes from the trial registers and seven studies from citation or manual search. After excluding duplicates, 10 272 articles were screened in the title/abstract stage, of which 195 articles were assessed in full text. We ultimately included 18 studies (with 21 reports) from this search and an additional 15 studies from the previous review (Appendix S2). A flow diagram of eligible article selection and reasons for exclusion is summarized in Fig. 1.

### Characteristics of the included studies

All 33 included studies were published from 2000 to 2024, with 23 studies (with 26 papers) published between 2018 and 2024. A total of 12 569 participants were included. The sample size of the studies ranged from 50 to 1800 patients. Included studies were primarily based on data from Europe (*n* = 17), followed by North America (*n* = 11), Asia (*n* = 3), and Oceania (*n* = 2). Most of the studies (21 studies) were individually randomized control trials (RCT). Ten studies were cluster-randomized trials (CRT), with two of them using a cluster stepped-wedge design. The remaining two were NRSI. Studies that reported our defined primary outcomes were included in the meta-analysis: 26 studies with 9449 participants.

Seventeen studies defined multimorbidity as having two or more chronic conditions, 14 studies as having three or more chronic conditions, and two studies did not report the number of chronic conditions required (details are summarized in Supplementary Table S1). The mean number of chronic health conditions per patient ranged from 2 to 12.7. The mean age of patients ranged from 50 to 82.5 years. Thirteen (39%) studies required participants to be aged 65 and above. The duration of intervention ranged from 1 to 24 months, with follow-up periods of up to 24 months. Usual primary healthcare received by the participants in their routine follow-up served as the comparator in all studies. Characteristics of the included studies are described in Supplementary Table S1. CC + SSM was the most studied intervention (*n* = 18 studies), followed by SSM (*n* = 9), and MM (*n* = 6). The results of the primary and secondary outcomes of all included studies are summarized in Supplementary Table S2.

### Risk of bias assessment

About 80% of the outcomes assessed using RoB 2 had low or some concern for risk of bias. The outcomes assessed to have high risk of bias were due to lack of clarity in the “Selection of the reported results” domain, “Deviations from intended interventions” domain, and “Missing outcome data” domain. The risk of bias for each outcome is interpreted in Supplementary Figure S1. Almost all the outcomes (95%) in CRT assessed by RoB 2 CRT had low risk of bias or some concern. Only three outcomes (5%) had high risk of bias. The risk of bias for each outcome is demonstrated in Supplementary file: Fig. 2. The outcomes in NRSI assessed using ROBINS-I all had moderate or serious risk of bias (Supplementary Figure S3).

**Figure 2.**
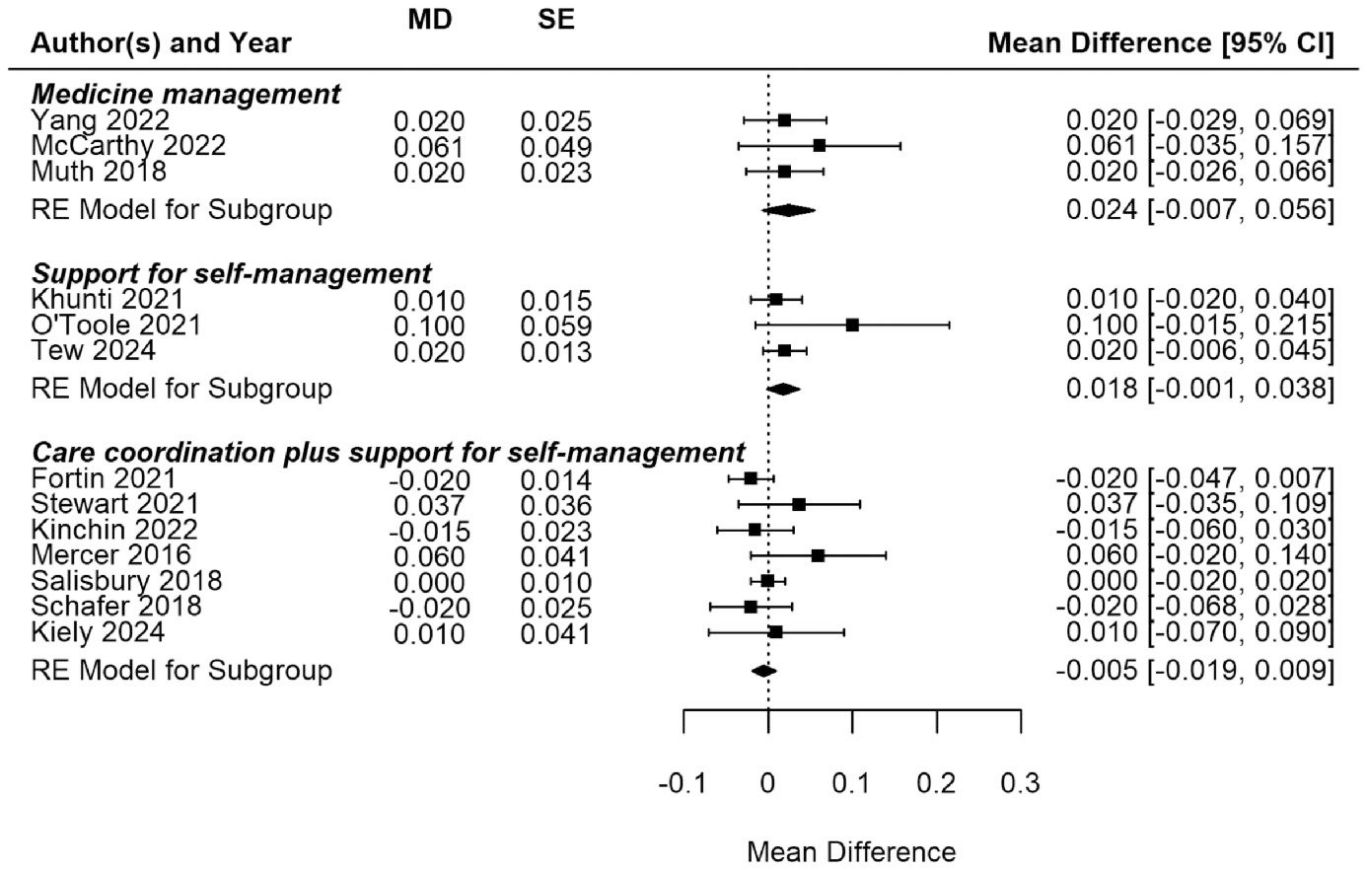
Meta-analysis of utility outcome for three intervention types.

### Effects of the interventions

In alignment with the previous review, we compiled the results for HRQoL (utility) for all three intervention types. Utility outcomes were reported by EQ-5D scores (EQ-5D-5L or EQ-5D-3L). Overall, MM and SSM interventions showed slightly better utility scores compared with usual care, but neither result was statistically significant (Fig. 2).

### Medicines management interventions

There were no statistically significant effects of MM on any of the review primary outcomes (Table 1).

**Table 1.** Summary of findings (medicine management interventions).

Outcomes	Treatment effects (95% CI)	No. of participants (studies)	Total heterogeneity, *I*^2^ (%)	Certainty of the evidence (GRADE)
**HRQoL**	MD unless stated otherwise			
Utility follow-up: range 3 months to 9 months	0.024 higher (0.007 lower to 0.056 higher)	801 (3 RCT)	0	⊕⊕◯◯ Low^a,d^
Physical Component Score follow-up: 15 months	0.250 lower (1.650 lower to 1.150 higher)	142 (1 RCT)	-	⊕⊕◯◯ Low^e^
Mental Component Score follow-up: 15 months	0.960 lower (2.740 lower to 0.820 higher)	142 (1 RCT)	-	⊕⊕◯◯ Low^e^
Other Quality of Life follow-up: 3 months	SMD 0.330 lower (0.668 lower to 0.009 higher)	136 (1 RCT)	-	⊕⊕◯◯ Low^a,d^
**Healthcare Utilization**				
Hospitalization	0.30 more (0.22 fewer to 0.83 more)	189 (2 RCT)	39	⊕⊕◯◯ Low^a,e^
Length of Hospital Stay	8.86 more (6.28 fewer to 24.08 more)	548 (3 RCT)	96	⊕◯◯◯ Very low^a,b,c,e^
Emergency Department Visits	0.08 fewer (0.87 fewer to 0.71 more)	495 (2 RCT)	71	⊕◯◯◯ Very low^a,b,c,e^
Outpatient Visits	0.11 fewer (0.85 fewer to 0.63 more)	495 (2 RCT)	0	⊕◯◯◯ Very low^a,e^
General Practitioner Visits	0.11 fewer (1.82 fewer to 1.59 more)	495 (2 RCT)	69	⊕◯◯◯ Very low^a,b,c,e^
**Healthcare Cost**				
Total Healthcare Costs	6 lower (168 lower to 157 higher)	539 (2 RCT)	0	⊕⊕◯◯ Low^a,d^
Inpatient Cost	1467 lower (5049 lower to 2116 higher)	403 (1 RCT)	-	⊕⊕◯◯ Low^e^
Outpatient Cost	90 higher (233 lower to 412 higher)	403 (1 RCT)	-	⊕⊕◯◯ Low^e^
Emergency Department Visits Cost	86 higher (39 lower to 211 higher)	403 (1 RCT)	-	⊕⊕◯◯ Low^e^
General Practice Visits Cost	70 higher (9 lower to 149 higher)	403 (1 RCT)	-	⊕⊕◯◯ Low^e^
Nurse Visits Cost	13 lower (60 lower to 34 higher)	403 (1 RCT)	-	⊕⊕◯◯ Low^e^

CI, confidence interval; HRQoL, health-related quality of life; MD, mean difference; RCT, randomized control trial; SMD, standardized mean difference.

^a^Downgraded due to risk of bias (High risk of bias found in Yang 2022 for this outcome due to the high number of missing data in the control group).

^b^Downgraded due to inconsistency (Heterogeneity).

^c^Downgraded due to indirectness (Outcomes for healthcare utilizations were collected in different ways).

^d^Downgraded due to imprecision (Small number of participants).

^e^Downgraded due to imprecision (Small number of participants and wide confidence interval).

### Self-management support interventions

There was no statistically significant effect on HRQoL outcomes, based on moderate, low or very low certainty of evidence for all measures. SSM resulted in fewer patients with hospitalization (OR 0.55, 95% CI 0.32 to 0.97, low certainty), although other measures of hospitalizations showed no clear evidence of reductions. SSM had more ED visits than usual care (MD 0.15/year, 95% CI 0.02 to 0.27, *I*^2^ = 0%, moderate certainty). SSM interventions were found to have lower total healthcare cost (MD −1803 USD/year, 95% CI −7214 to 3,608, *I*^2^ 65%, low certainty) and all other healthcare costs, except those for GP visits, but these estimates were imprecise and not statistically significant for any cost categories except medication costs (MD −110 USD/year, 95% CI −151 to −68, moderate certainty). A summary of findings for all the review primary outcomes for SSM interventions compared with usual care is shown in Table 2.

**Table 2.** Summary of findings (support for self-management interventions).

Outcomes	Treatment effects (95% CI)	No. of participants (studies)	Total heterogeneity, *I*^2^ (%)	Certainty of the evidence (GRADE)
**HRQoL**	MD unless stated otherwise			
Utility follow-up: range 6 months to 12 months	0.018 higher (0.001 lower to 0.038 higher)	777 (3 RCT)	2	⊕⊕⊕◯ Moderate^e^
Physical Component Score follow-up: range 6 months to 12 months	1.605 higher (3.420 lower to 6.630 higher)	250 (2 RCT)	78	⊕◯◯◯ Very low^a,b,e^
Mental Component Score follow-up: range 6 months to 12 months	1.969 higher (1.774 lower to 5.711 higher)	250 (2 RCT)	50	⊕⊕◯◯ Low^a,e^
Other Quality of Life follow-up: range 6 months to 24 months	SMD 0.178 higher (0.087 lower to 0.442 higher)	1072 (5 RCT)	75	⊕◯◯◯ Very low^b,c,e^
**Healthcare Utilization**				
Hospitalization	0.04 fewer (0.30 fewer to 0.22 more)	648 (4 RCT)	46	⊕⊕◯◯ Low^b,d^
Number of Patients with Hospitalization	OR 0.55* (0.32 to 0.97)	229 (1 RCT)	-	⊕⊕◯◯ Low^f^
Length of Hospital Stay	2.12 fewer (7.18 fewer to 2.93 more)	508 (2 RCT)	67	⊕⊕⊕◯ Moderate^e^
Outpatient Visits	1.55 fewer (4.86 fewer to 1.76 more)	503 (2 RCT)	91	⊕⊕⊕◯ Moderate^e^
Emergency Department Visits	0.15 more* (0.02 more to 0.27 more)	764 (3 RCT)	0	⊕⊕⊕◯ Moderate^e^
General Practitioner Visits	0.24 more (0.40 fewer to 0.89 more)	808 (4 RCT)	21	⊕⊕⊕◯ Moderate^e^
Nurse Visits	0.11 fewer (0.43 fewer to 0.22 more)	414 (2 RCT)	0	⊕⊕◯◯ Low^d,e^
**Healthcare Cost**				
Total Healthcare Costs	1803 lower (7214 lower to 3608 higher)	603 (2 RCT)	65	⊕⊕◯◯ Low^f^
Inpatient Cost	1873 lower (6774 lower to 3027 higher)	603 (2 RCT)	61	⊕⊕◯◯ Low^f^
Outpatient Cost	312 lower (924 lower to 300 higher)	603 (2 RCT)	90	⊕◯◯◯ Very low^b,f^
Emergency Department Visits Cost	0.4 lower (179 lower to 180 higher)	603 (2 RCT)	0	⊕⊕◯◯ Low^f^
General Practice Visits Cost	11 higher (13 lower to 34 higher)	603 (2 RCT)	0	⊕⊕⊕◯ Moderate^e^
Nurse Visits Cost	4 lower (13 lower to 5 higher)	454 (1 RCT)	-	⊕⊕⊕◯ Moderate^e^
Medication Cost	110 lower (151 lower to 68 lower)	454 (1 RCT)	-	⊕⊕⊕◯ Moderate^e^

^*^Statistically significant; CI, confidence interval; HRQoL, health-related quality of life; MD, mean difference; OR, odds ratio; RCT, randomized control trial; SMD, standardized mean difference.

^a^Downgraded due to risk of bias (High risk of bias).

^b^Downgraded due to inconsistency (Heterogeneity).

^c^Downgraded due to indirectness (Outcomes were assessed by different tools).

^d^Downgraded due to indirectness (Outcomes for healthcare utilizations were collected in different ways).

^e^Downgraded due to imprecision (Small number of participants).

^f^Downgraded due to imprecision (Small number of participants and wide confidence interval).

### Care-coordination plus self-management support interventions

Compared with usual care, CC + SSM showed a small improvement in HRQoL when measured by SF-12 PCS scores (MD 0.779, 95% CI 0.200 to 1.358, *I*^2^ 0%, moderate certainty), but no significant difference in other HRQoL outcomes. CC + SSM interventions reduced the number of patients with hospitalization (OR 0.56, 95% CI 0.39 to 0.81, *I*^2^ 0%, moderate certainty) and length of hospital stays (MD 0.99 days/year, 95% CI −1.88 to −0.10, *I*^2^ 36%, low certainty), increased the number of outpatient visits (MD 0.38/year, 95% CI 0.17 to 0.59, *I*^2^ 9%, moderate certainty) and GP visits (MD 3.69/year, 95% CI 0.41 to 6.96, *I*^2^ 99%, very low certainty). CC + SSM resulted in higher healthcare costs for most cost categories, but these estimates were based on very few studies and were not statistically significant. A summary of findings for all the review primary outcomes for CC + SSM interventions compared with usual care is shown in Table 3.

**Table 3.** Summary of findings (care coordination plus support for self-management interventions).

Outcomes	Treatment effects (95% CI)	No. of participants (studies)	Total heterogeneity, *I*^2^ (%)	Certainty of the evidence (GRADE)
**HRQoL**	MD unless stated otherwise			
Utility follow-up: range 1 months to 15 months	0.005 lower (0.019 lower to 0.009 higher)	3226 (7 RCT)	1	⊕⊕⊕◯ Moderate^f^
Physical Component Score follow-up: range 4 months to 12 months	0.779 higher* (0.200 higher to 1.358 higher)	1088 (6 RCT)	0	⊕⊕⊕◯ Moderate^f^
Mental Component Score follow-up: range 4 months to 12 months	0.525 higher (0.072 lower to 1.123 higher)	1088 (6 RCT)	0	⊕⊕⊕◯ Moderate^f^
Other Quality of Life follow-up: range 1 months to 12 months	SMD 0.012 higher (0.152 lower to 0.176 higher)	759 (3 RCT)	21	⊕⊕◯◯ Low^b,d^
**Healthcare Utilization**				
Hospitalization	0.01 fewer (0.06 fewer to 0.04 more)	4111 (6 RCT + 1 NRSI)	0	⊕⊕◯◯ Low^b^,^c^
Number of Patients with Hospitalization	OR 0.56* (0.39 to 0.81)	497 (2 RCT)	0	⊕⊕⊕◯ Moderate^f^
Length of Hospital Stay	0.99 fewer* (1.88 fewer to 0.10 fewer)	2013 (4 RCT)	36	⊕⊕◯◯ Low^b^,^f^
Number of Patients with Rehospitalization	OR 0.55 (0.25 to 1.19)	543 (1 RCT)	-	⊕⊕◯◯ Low^e^
Outpatient Visits	0.38 more* (0.17 more to 0.59 more)	3222 (4 RCT)	9	⊕⊕⊕◯ Moderate^f^
Emergency Department Visits	0.25 fewer (0.59 fewer to 0.10 more)	2508 (6 RCT + 1 NRSI)	71	⊕◯◯◯ Very low^f^,^c,f^
Number of Patients with Emergency Department Visits	OR 1.40 (0.76 to 2.56)	182 (2 RCT)	0	⊕⊕◯◯ Low^e^
General Practitioner Visits	3.69 more* (0.41 more to 6.96 more)	4127 (5 RCT + 1 NRSI)	99	⊕◯◯◯ Very low^a^,^b^,^c^
Nurse Visits	0.54 more (2.32 fewer to 3.40 more)	2514 (2 RCT + 1 NRSI)	95	⊕◯◯◯ Very low^a^,^c,f^
Allied Health Visits	0.37 fewer (2.71 fewer to 1.98 more)	924 (2 RCT)	68	⊕⊕◯◯ Low^b^,^f^
Social Support	4.71 more (2.94 fewer to 12.35 more)	1329 (3 RCT)	71	⊕◯◯◯ Very low^a^,^e^
Ambulance Services Use	0.08 more (0.07 fewer to 0.22 more)	320 (1 RCT)	-	⊕⊕◯◯ Low^e^
**Healthcare Cost**				
Total Healthcare Costs	1302 higher (150 lower to 2755 higher)	592 (3 RCT)	0	⊕◯◯◯ Very low^a,e^
Inpatient Cost	474 fewer (1039 lower to 90 higher)	352 (2 RCT)	0	⊕⊕◯◯ Low^e^
Outpatient Cost	13 higher (81 lower to 108 higher)	32 (1 RCT)	-	⊕⊕◯◯ Low^e^
Emergency Department Cost	77 higher (57 lower to 212 higher)	352 (2 RCT)	0	⊕⊕◯◯ Low^e^
General Practitioner Visits Cost	258 higher (63 lower to 580 higher)	32 (1 RCT)	-	⊕⊕◯◯ Low^e^
Allied Health Visits Cost	215 higher (53 lower to 484 higher)	320 (1 RCT)	-	⊕⊕◯◯ Low^e^
Social Support Cost	76 higher (257 lower to 409 higher)	320 (1 RCT)	-	⊕⊕◯◯ Low^e^
Ambulance Cost	36 higher (40 lower to 112 higher)	352 (2 RCT)	0	⊕⊕◯◯ Low^e^
Medication cost	292 higher (631 lower to 1215 higher)	32 (1 RCT)	-	⊕⊕◯◯ Low^e^

^*^Statistically significant; CI, confidence interval; HRQoL, health-related quality of life; MD, mean difference; NRSI, non-randomized studies of interventions; OR, odds ratio; RCT, randomized control trial; SMD, standardized mean difference.

^a^Downgraded due to inconsistency (Heterogeneity).

^b^Downgraded due to indirectness (Outcomes for healthcare utilizations were collected in different ways).

^c^Downgraded due to indirectness (Both RCTs and NRSI were included in the analysis).

^d^Downgraded due to indirectness (Outcomes were assessed by different tools).

^e^Downgraded due to imprecision (Small number of participants and wide confidence interval).

^f^Downgraded due to imprecision (Small number of participants).

### Secondary outcomes

Studies of MM interventions generally focused on medicines outcomes, which were secondary outcomes for this review. Several of these showed improvements in medication adherence [28], medication appropriateness [29], and reduction of polypharmacy [30]. Several studies in SSM and CC + SSM interventions showed improvements in health behaviours [31, 32], and psychosocial outcomes [33, 34]. Results for all secondary outcomes are reported in Supplementary Table S3.

### Sensitivity analysis for risk of bias and types of study design

One study [28] of MM interventions was considered to be at high risk of bias. When excluding this study from the meta-analysis, two studies [30, 35] remained for the health utility and length of hospital stay outcomes, and one small study for the hospitalization [35], ED visits [30] and outpatient visits [30] outcomes. The estimate of the improvement in health utility scores and the treatment effect for healthcare utilization outcomes remained similar to that in the main analysis.

There was also one study [36] of SSM interventions considered to be at high risk of bias. When excluding this study, a single small study [37] remained for the SF-12 PCS and MCS outcomes. The improvement in PCS scores was larger than in the main analysis, and statistically significant (MD 4.420, 95% CI 0.445 to 8.395), while there was no significant effect on MCS scores in either the main analysis or the sensitivity analysis.

One study [38] of CC + SSM interventions was considered to be at high risk of bias. When excluding this study, five studies [32, 39–42] remained for SF-12 PCS and MCS scores and two [43, 44] for other HRQoL outcomes. The results for all of these outcomes remained similar to the main analysis, but the effect on PCS scores was no longer statistically significant (MD 0.802, 95% CI −0.877 to 2.482, *I*^2^ 32%).

When excluding the a non-randomized (quasi-experimental) study [45] of CC + SSM interventions, the results remained generally similar, although there was no longer a decrease in ED visits (MD 0.02 visits/year, 95% CI −0.15 to 0.20, *I*^2^ 0%) and the increase in GP visits was no longer statistically significant (MD 3.26 visits/year, 95% CI −0.64 to 7.16, *I*^2^ 100%). Results for all sensitivity analyses are reported in Supplementary Table S4.

## Discussion

In this update of Smith *et al*. [2], we systematically reviewed 33 studies of interventions for multimorbidity. In addition to HRQoL outcomes, we focused on healthcare utilization and healthcare costs as our primary outcomes. We found some suggestion of better HRQoL and reduced healthcare utilization, especially reduction of hospitalizations, in intervention groups as compared to usual care. Nevertheless, there was little evidence of consistently improved health outcomes or healthcare utilization measures across the included interventions. Our review further reveals a paucity of multimorbidity literature reporting on healthcare costs.

### Medicine management interventions

Most studies of MM interventions focused on medicine outcomes (e.g. adherence, appropriateness, polypharmacy), and several found improvements in these outcomes. However, we found little evidence of benefits for our primary outcomes of healthcare use, healthcare costs, and HRQoL in these interventions over usual care.

### Self-management support interventions

We saw slightly better outcomes across all of the HRQoL measures with SSM interventions, although these improvements were not statistically significant compared with usual care. SSM interventions were associated with reduced odds of patient hospitalization, consistent with a systematic review that found that self-management support interventions can reduce health service utilization without compromising patient health outcomes [46]. However, this finding was based on one study with small sample size and is of low certainty; other measures of hospitalization did not show significant evidence of treatment effects. There was also some suggestion of lower healthcare costs associated with SSM interventions, but these were of low certainty of evidence and were generally not statistically significant.

### Care-coordination plus self-management support interventions

CC + SSM interventions showed some better HRQoL and healthcare utilization outcomes especially in reducing hospitalization. This is consistent with previous studies reporting that care coordination may assist people living with multimorbidity to better manage their health conditions and reduce potentially preventable hospitalizations [47–49]. In contrast, CC + SSM interventions were associated with more outpatient and GP visits. This could be due to the nature of the interventions where this type of patient-centred interventions requires a close connection and contact between patients and multiple healthcare professionals [50]. By increasing the contact between patients and appropriate health services and healthcare providers, we should see more use of the health services (for instance more outpatient and GP visits summarized in this review), which likely led to the finding of fewer hospitalizations.

Overall, most of the outcomes had small and uncertain treatment effects. This could be due to the short duration of treatment or follow-up period in the included studies, where the duration of intervention and follow-up period were generally ranged from 1 month to 24 months. Multimorbidity involves multiple long-term health conditions which require management and monitoring over a long period of time, so short-duration trials may be unable to identify benefits of interventions [51]. Providing treatment for a multimorbid patient over a long period of time is beneficial for understanding the patient's history of diseases, illness experience, and life circumstances, and allows GPs to provide better long-term follow-up, monitor the safety of any medication changes, and observe patients for both adverse reactions and therapeutic benefits [52].

Additionally, small treatment effects are a common feature in most evaluations of multimorbidity interventions [13, 53, 54], particularly for broad outcome measures that may not detect subtle changes in a highly heterogeneous patient population with varying symptoms, conditions, and medications especially in those with polypharmacy [55].

Since multimorbidity leads to increased healthcare utilization and primary care costs [56], we included healthcare cost as one of the primary outcomes in this review. Nevertheless, only a handful of studies (*n* = 7) reported on costs associated with the healthcare resources used. All of these studies were economic evaluations conducted from the public health system perspective except Yang 2022 [28], which was a clinical trial that reported only a selection of costs relevant to the study. Due to the low number of studies involved in the meta-analysis, the results are not robust enough to draw a conclusion about the effects of the interventions on healthcare cost [57].

The number of clinical trials on multimorbidity has increased enormously within the recent 5 years. Given that new data contained in recent studies could change the conclusion of existing reviews, updating the review is necessary to reflect emerging evidence [58]. A strength of this review is that we identified and included NRSI in addition to randomized trials. We identified 18 studies for our review that were not included in the previous review, providing additional evidence on effects on healthcare utilization and costs as well as HRQoL, which was the primary outcome reported in the earlier review. However, including NRSI might increase the risk of bias in the estimates of the treatment effects. Thus, we performed sensitivity analysis by removing NRSI. Most of the findings of the primary analysis were robust to the exclusion of NRSI.

### Future research direction for multimorbidity

Since multimorbidity involves complex management, future research on multimorbidity should focus on prospective longitudinal studies with repeat measures over long follow-up periods that could focus on understanding the disease causal pathways as well as the accumulation of treatment effects of the interventions used, which will in turn better inform clinical practice and policy implication [59, 60]. Additionally, future research should collect and report data on healthcare resources use and costs to enable economic evaluation of the interventions used to improve health outcomes for patients with multimorbidity.

## Conclusions

In conclusion, multimorbidity presents a complex challenge to both patients and healthcare system. As the prevalence of multimorbidity continues to rise, it becomes increasingly critical to develop and adopt a comprehensive strategy that is evidence-based and integrates medical, psychological, and social support to improve patient outcomes and quality of life while controlling healthcare spending. This review found limited evidence for the effectiveness of interventions for the management of multimorbidity in primary and community care settings, with a particular paucity of reporting of healthcare costs.

## Supplementary Material

cmaf085_Supplementary_Data

## Data Availability

The data underlying this article are available in the article and in its online Supplementary material.
