# Supplementary material for: Effectiveness of interventions for the management of multimorbidity in primary care and community settings: systematic review and meta-analysis
Source: Fam Pract. 2025 Nov 11;42(6):cmaf085. doi: 10.1093/fampra/cmaf085 (PMC12602864; doi:10.1093/fampra/cmaf085)
Supplement: cmaf085_Supplementary_Data [file cmaf085_supplementary_data.zip › Supplementary Material.pdf]

Appendix 1: Search Strategies

Medline (OVID)

No.	Search terms
1	comorbidity/
2	multimorbidity/
3	chronic disease/
4	(comorbid* or co-morbid*).ti,ab,kf.
5	(multimorbid* or multi-morbid*).ti,ab,kf.
6	(multidisease? or multicondition? or ((multi or multiple) adj2 (morbid* or ill* or disease? or condition? or syndrom* or disorder?))).ti,ab,kf.
7	(chronic* adj (disease? or ill* or care or condition? or disorder* or health* or medication* or syndrom* or symptom*)).ti,ab,kf.
8	or/1-7
9	exp primary health care/
10	family practice/
11	physicians, primary care/
12	general practice/
13	physicians, family/
14	general practitioners/
15	exp outpatient clinics, hospital/
16	ambulatory care/
17	exp ambulatory care facilities/
18	exp community health services/
19	exp community health centers/
20	((primary or communit*) adj5 (care or health*)).ti,ab,kf.
21	(family practi* or family doctor* or family physician* or gp* or general practi*).ti,ab,kf.
22	((outpatient? or ambulatory) adj2 (care or healthcare or clinic? or service? or facilit*)).ti,ab,kf.
23	(community adj2 (site? or practice? or clinic? or based or facilit*)).ti,ab,kf.
24	or/9-23

25	((organisation* or organization*) adj2 (intervention* or program*)).ti,ab,kf.
26	(self care or self management).ti,ab,kf.
27	(case management or care management).ti,ab,kf.
28	((integrat* or coordinated or co-ordinated or collaborat*) adj2 care).ti,ab,kf.
29	((financ* or money or monetary or cash) adj2 incentiv*).ti,ab,kf.
30	(patient adj2 educat*).ti,ab,kf.
31	((provider? or physician? or doctor? or nurse or health or healthcare) adj2 educat*).ti,ab,kf.
32	((multicomponent or multi-component or multifacet* or multi-facet* or multidisciplinary or multi-disciplinary or interdisciplinary or inter-disciplinary) adj2 (care or team? or intervention? or program*)).ti,ab,kf.
33	(care plan? or guided care or (personal* adj care)).ti,ab,kf.
34	(home adj2 (care or intervention or program)).ti,ab,kf.
35	exp self care/
36	self-management/
37	case management/
38	case managers/
39	"delivery of health care, integrated"/
40	patient care planning/
41	exp insurance, health, reimbursement/
42	patient care team/
43	home care services/
44	patient education as topic/
45	exp education professional/
46	exp inservice training/
47	or/25-46
48	randomized controlled trial.pt.
49	controlled clinical trial.pt.
50	multicenter study.pt.
51	pragmatic clinical trial.pt.
52	(randomis* or randomiz* or randomly).ti,ab.

53	groups.ab.
54	(trial or multicenter or multi center or multicentre or multi centre).ti.
55	(intervention? or effect? or impact? or controlled or control group? or (before adj5 after) or (pre adj5 post) or ((pretest or pre test) and (posttest or post test)) or quasiexperiment* or quasi experiment* or pseudo experiment* or pseudoexperiment* or evaluat* or time series or time point? or repeated measur*).ti,ab.
56	non-randomized controlled trials as topic/
57	interrupted time series analysis/
58	controlled before-after studies/
59	or/48-58
60	exp animals/
61	humans/
62	60 not (60 and 61)
63	review.pt.
64	meta analysis.pt.
65	news.pt.
66	comment.pt.
67	editorial.pt.
68	cochrane database of systematic reviews.jn.
69	comment on.cm.
70	(systematic review or literature review).ti.
71	or/62-70
72	59 not 71
73	8 and 24 and 47 and 72
74	limit 73 to yr="2019 -Current"

Embase (OVID)

No.	Search terms
1	comorbidity/
2	multiple chronic conditions/

3	chronic disease/
4	(comorbid* or co-morbid*).ti,ab,kw.
5	(multimorbid* or multi-morbid*).ti,ab,kw.
6	(multidisease? or multicondition? or ((multi or multiple) adj2 (morbid* or ill* or disease? or condition? or syndrom* or disorder?))).ti,ab,kw.
7	(chronic* adj (disease? or ill* or care or condition? or disorder* or health* or medication* or syndrom* or symptom*)).ti,ab,kw.
8	or/1-7
9	exp primary health care/
10	general practice/
11	general practitioner/
12	outpatient department/
13	outpatient care/
14	ambulatory care/
15	community care/
16	((primary or communit*) adj5 (care or health*)).ti,ab,kw.
17	(family practi* or family doctor* or family physician* or gp* or general practi*).ti,ab,kw.
18	((outpatient? or ambulatory) adj2 (care or healthcare or clinic? or service? or facilit*)).ti,ab,kw.
19	(community adj2 (site? or practice? or clinic? or based or facilit*)).ti,ab,kw.
20	or/9-19
21	((organisation* or organization*) adj2 (intervention* or program*)).ti,ab,kw.
22	(self care or self management).ti,ab,kw.
23	(case management or care management).ti,ab,kw.
24	((integrat* or coordinated or co-ordinated or collaborat*) adj2 care).ti,ab,kw.
25	((financ* or money or monetary or cash) adj2 incentiv*).ti,ab,kw.
26	(patient adj2 educat*).ti,ab,kw.
27	((provider? or physician? or doctor? or nurse or health or healthcare) adj2 educat*).ti,ab,kw.
28	((multicomponent or multi-component or multifacet* or multi-facet* or multidisciplinary or multi-disciplinary or interdisciplinary or inter-disciplinary) adj2 (care or team? or intervention? or program*)).ti,ab,kw.
29	(care plan? or guided care or (personal* adj care)).ti,ab,kw.

30	(home adj2 (care or intervention or program)).ti,ab,kw.
31	exp self care/
32	case management/
33	case manager/
34	integrated health care system/
35	patient care planning/
36	reimbursement/
37	exp home care/
38	patient education/
39	education program/
40	in service training/
41	continuing education/
42	vocational education/
43	or/21-42
44	randomized controlled trial/
45	controlled clinical trial/
46	quasi experimental study/
47	pretest posttest control group design/
48	time series analysis/
49	experimental design/
50	multicenter study/
51	(randomis* or randomiz* or randomly).ti,ab.
52	groups.ab.
53	(trial or multicentre or multicenter or multi centre or multi center).ti.
54	(intervention? or effect? or impact? or controlled or control group? or (before adj5 after) or (pre adj5 post) or ((pretest or pre test) and (posttest or post test)) or quasiexperiment* or quasi experiment* or pseudo experiment* or pseudoexperiment* or evaluat* or time series or time point? or repeated measur*).ti,ab.
55	or/44-54
56	(systematic review or literature review).ti.
57	"cochrane database of systematic reviews".jn.

58	exp animals/ or exp invertebrate/ or animal experiment/ or animal model/ or animal tissue/ or animal cell/ or nonhuman/
59	human/ or normal human/ or human cell/
60	58 not (58 and 59)
61	56 or 57 or 60
62	55 not 61
63	8 and 20 and 43 and 62
64	limit 63 to yr="2019 -Current"

The Cochrane Library

No.	Search terms
#1	[mh comorbidity]
#2	[mh multimorbidity]
#3	[mh "chronic disease"]
#4	(comorbid* or co-morbid*):ti,ab
#5	(multimorbid* or multi-morbid*):ti,ab
#6	(multidisease? or multicondition? or ((multi or multiple) near/2 (morbid* or ill* or disease? or condition? or syndrom* or disorder?))):ti,ab
#7	(chronic* next (disease? or ill* or care or condition? or disorder* or health* or medication* or syndrom* or symptom*)):ti,ab
#8	#1 OR #2 OR #3 OR #4 OR #5 OR #6 OR #7
#9	[mh "primary health care"]
#10	[mh "family practice"]
#11	[mh "physicians, primary care"]
#12	[mh "general practice"]
#13	[mh "physicians, family"]
#14	[mh "general practitioners"]
#15	[mh "outpatient clinics, hospital"]
#16	[mh "ambulatory care"]
#17	[mh "ambulatory care facilities"]

#18	[mh "community health services"]
#19	[mh "community health centers"]
#20	((primary or communit*) near/5 (care or health*)):ti,ab
#21	(family next practi* or family next doctor* or family next physician* or gp* or general next practi*):ti,ab
#22	((outpatient? or ambulatory) near/2 (care or healthcare or clinic? or service? or facilit*)):ti,ab
#23	(community near/2 (site? or practice? or clinic? or based or facilit*)):ti,ab
#24	#9 OR #10 OR #11 OR #12 OR #13 OR #14 OR #15 OR #16 OR #17 OR #18 OR #19 OR #20 OR #21 OR #22 OR #23
#25	((organisation* or organization*) near/2 (intervention* or program*)):ti,ab
#26	(self next care or self next management):ti,ab
#27	(case next management or care next management):ti,ab
#28	((integrat* or coordinated or co-ordinated or collaborat*) near/2 care):ti,ab
#29	((financ* or money or monetary or cash) near/2 incentiv*):ti,ab
#30	(patient near/2 educat*):ti,ab
#31	((provider? or physician? or doctor? or nurse or health or healthcare) near/2 educat*):ti,ab
#32	((multicomponent or multi-component or multifacet* or multi-facet* or multidisciplinary or multi-disciplinary or interdisciplinary or inter-disciplinary) near/2 (care or team? or intervention? or program*)):ti,ab
#33	(care next plan? or guided next care or (personal* next care)):ti,ab
#34	(home near/2 (care or intervention or program)):ti,ab
#35	[mh "self care"]
#36	[mh self-management]
#37	[mh "case management"]
#38	[mh "case managers"]
#39	[mh "delivery of health care, integrated"]
#40	[mh "patient care planning"]
#41	[mh "insurance, health, reimbursement"]
#42	[mh "patient care team"]
#43	[mh "home care services"]
#44	[mh "patient education as topic"]

#45	education professional
#46	[mh "inservice training"]
#47	#25 OR #26 OR #27 OR #28 OR #29 OR #30 OR #31 OR #32 OR #33 OR #34 OR #35 OR #36 OR #37 OR #38 OR #39 OR #40 OR #41 OR #42 OR #43 OR #44 OR #45 OR #46
#48	#8 and #24 and #47
	with Cochrane Library publication date from Jan 2019 to Oct 2023

CINAHL (EBSCO)

No.	Search terms
S1	(MH "Comorbidity")
S2	(MH "Chronic Disease")
S3	(comorbid* or co-morbid*)
S4	(multimorbid* or multi-morbid*)
S5	(multidisease? or multicondition? or ((multi or multiple) N2 (morbid* or ill* or disease? or condition? or syndrom* or disorder?)))
S6	(chronic* N0 (disease? or ill* or care or condition? or disorder* or health* or medication* or syndrom* or symptom*))
S7	S1 OR S2 OR S3 OR S4 OR S5 OR S6
S8	(MH "Primary Health Care")
S9	(MH "Family Practice")
S10	(MH "Physicians, Family")
S11	(MH "Ambulatory Care Facilities")
S12	(MH "Outpatient Service")
S13	(MH "Ambulatory Care")
S14	(MH "Community Health Services+")
S15	(MH "Community Health Centers+")
S16	(primary or communit*) N5 (care or health*)
S17	family practi* or family doctor* or family physician* or gp* or general practi*
S18	(outpatient? or ambulatory) N2 (care or healthcare or clinic? or service? or facilit*)
S19	(community N2 (site? or practice? or clinic? or based or facilit*))

S20	S8 OR S9 OR S10 OR S11 OR S12 OR S13 OR S14 OR S15 OR S16 OR S17 OR S18 OR S19
S21	((organisation* or organization*) N2 (intervention* or program*))
S22	(self care or self management)
S23	(case management or care management)
S24	((integrat* or coordinated or co-ordinated or collaborat*) N2 care)
S25	((financ* or money or monetary or cash) N2 incentiv*)
S26	(patient N2 educat*)
S27	((provider? or physician? or doctor? or nurse or health or healthcare) N2 educat*)
S28	((multicomponent or multi-component or multifacet* or multi-facet* or multidisciplinary or multi-disciplinary or interdisciplinary or inter-disciplinary) N2 (care or team? or intervention? or program*))
S29	(care plan? or guided care or (personal* care))
S30	(home N2 (care or intervention or program))
S31	(MH "Self Care+")
S32	(MH "Case Management")
S33	(MH "Case Managers")
S34	(MH "Health Care Delivery, Integrated")
S35	(MH "Multidisciplinary Care Team")
S36	(MH "Patient Care Plans+")
S37	(MH "Insurance, Health, Reimbursement+")
S38	(MH "Home Health Care+")
S39	(MH "Patient Education+")
S40	(MH "Education+")
S41	S21 OR S22 OR S23 OR S24 OR S25 OR S26 OR S27 OR S28 OR S29 OR S30 OR S31 OR S32 OR S33 OR S34 OR S35 OR S36 OR S37 OR S38 OR S39 OR S40
S42	S7 AND S20 AND S41
S43	PT randomized controlled trial
S44	PT clinical trial
S45	PT research
S46	(MH "Randomized Controlled Trials")
S47	(MH "Clinical Trials")

S48	(MH "Intervention Trials")
S49	(MH "Nonrandomized Trials")
S50	(MH "Experimental Studies")
S51	(MH "Pretest-Posttest Design+")
S52	(MH "Quasi-Experimental Studies+")
S53	(MH "Multicenter Studies")
S54	(MH "Health Services Research")
S55	TI (randomis* or randomiz* or randomly) OR AB (randomis* or randomiz* or randomly)
S56	TI (trial or effect* or impact* or intervention* or before N5 after or pre N5 post or ((pretest or "pre test") and (posttest or "post test")) or quasiexperiment* or quasi W0 experiment* or pseudo experiment* or pseudoexperiment* or evaluat* or "time series" or time W0 point* or repeated W0 measur*) OR AB (trial or effect* or impact* or intervention* or before N5 after or pre N5 post or ((pretest or "pre test") and (posttest or "post test")) or quasiexperiment* or quasi W0 experiment* or pseudo experiment* or pseudoexperiment* or evaluat* or "time series" or time W0 point* or repeated W0 measur*)
S57	S43 OR S44 OR S45 OR S46 OR S47 OR S48 OR S49 OR S50 OR S51 OR S52 OR S53 OR S54 OR S55 OR S56
S58	S42 AND S57
S59	S58 Limiters - Publication Date: 20190101-20231031; English Language; Exclude MEDLINE records

**ClinicalTrials.gov**
multimorbidity OR comorbidity
**WHO International Clinical Trials Registry Platform (ICTRP)**
multimorbidity OR comorbidity

Appendix 2: List of Included studies in systematic review

Studies included in current systematic review			
**Author(s)**	**Year**	**Title**	**DOI**
Mazya AL, Garvin P, Ekdahl AW.	2019	Outpatient comprehensive geriatric assessment: effects on frailty and mortality in old people with multimorbidity and high health care utilization	10.1007/s40520-018-1004-z
Fisher K, Markle-Reid M, Ploeg J, et al.	2020	Self-management program versus usual care for community-dwelling older adults with multimorbidity: A pragmatic randomized controlled trial in Ontario, Canada	10.1177/2235042X20963390
Mateo-Abad M, Fullaondo A, Merino M, et al.	2020	Impact Assessment of an Innovative Integrated Care Model for Older Complex Patients with Multimorbidity: The CareWell Project	10.5334/ijic.4711
Shang-Lin C, Chien-Lung S, Liang-Cheng C, Yi-Pang L, Chueh-Ho L and Chia-Huei L.	2020	Effectiveness of a Home-Based Telehealth Exercise Training Program for Patients With Cardiometabolic Multimorbidity: A Randomized Controlled Trial	10.1097/JCN.0000000000000693
Fortin M, Stewart M, Ngangue P, et al.	2021	Scaling Up Patient-Centered Interdisciplinary Care for Multimorbidity: A Pragmatic Mixed-Methods Randomized Controlled Trial	10.1370/afm.2650
Hong K, Sulo S, Wang W, et al.	2021	Nutrition Care for Poorly Nourished Outpatients Reduces Resource Use and Lowers Costs	10.1177/21501327211017014
Khunti K, Highton PJ, Waheed G, et al.	2021	Promoting physical activity with self-management support for those with multimorbidity: a randomised controlled trial	10.3399/BJGP.2021.0172
Lear SA, Norena M, Banner D, et al.	2021	Assessment of an Interactive Digital Health-Based Self-management Program to Reduce Hospitalizations Among Patients With Multiple Chronic Diseases: A Randomized Clinical Trial	10.1001/jamanetworkopen.2021.40591
Lee WJ, Peng LN, Lin CH, et al.	2021	Effects of incorporating multidomain interventions into integrated primary care on quality of life: a randomised controlled trial	10.1016/S2666-7568(21)00248-8
M. Stewart, M. Fortin, J. B. Brown, et al.	2021	Patient-centred innovation for multimorbidity care: a mixed-methods, randomised trial and qualitative study of the patients' experience	10.3399/bjgp21X714293
O'Toole L, Connolly D, Boland F, Smith SM.	2021	Effect of the OPTIMAL programme on self-management of multimorbidity in primary care: a randomised controlled trial	10.3399/bjgp20X714185
Gillespie P, Hobbins A, O'Toole L, Connolly D, Boland F, Smith SM.	2022	Cost-effectiveness of an occupational therapy-led self-management support programme for multimorbidity in primary care	10.1093/fampra/cmac006
Kinchin I, Kelley S, Meshcheriakova E, et al.	2022	Cost-effectiveness of a community-based integrated care model compared with usual care for older adults with complex needs: a stepped-wedge cluster-randomised trial	10.1136/ihj-2022-000137
Sturm N, Krisam J, Szecsenyi J, et al.	2022	Spirituality, Self-Care, and Social Activity in the Primary Medical Care of Elderly Patients	10.3238/arztebl.m2022.0078
Yang C, Lee DTF, Wang X, Chair SY.	2022	Effects of a nurse-led medication self-management intervention on medication adherence and health outcomes in older people with multimorbidity: A randomised controlled trial	10.1016/j.ijnurstu.2022.104314
McCarthy C, Clyne B, Boland F, et al.	2022	GP-delivered medication review of polypharmacy, deprescribing, and patient priorities in older people with multimorbidity in Irish primary care (SPPiRE Study): A cluster randomised controlled trial	10.1371/journal.pmed.1003862

Gillespie, P., Moriarty, F., Smith, S.M. *et al.*	2025	Cost effectiveness of a GP delivered medication review to reduce polypharmacy and potentially inappropriate prescribing in older patients with multimorbidity in Irish primary care: the SPPiRE cluster randomised controlled trial.	10.1007/s10198-024-01718-7
Chan B, Edwards ST, Srikanth P, et al.	2023	Ambulatory Intensive Care for Medically Complex Patients at a Health Care Clinic for Individuals Experiencing Homelessness: The SUMMIT Randomized Clinical Trial	10.1001/jamanetworkopen.2023.42012
Kiely B, Hobbins A, Boland F, et al.	2024	An exploratory randomised trial investigating feasibility, potential impact and cost effectiveness of link workers for people living with multimorbidity attending general practices in deprived urban communities	10.1186/s12875-024-02482-6
Tew GA, Wiley L, Ward L, et al.	2024	Chair-based yoga programme for older adults with multimorbidity: RCT with embedded economic and process evaluations	10.3310/KPGN4216
**Studies included in previous systematic review**			
**Author(s)**	**Year**	**Title**	**DOI**
Sommers LS, Marton KI, Barbaccia JC, Randolph J.	2000	Physician, nurse, and social worker collaboration in primary care for chronically ill seniors	10.1001/archinte.160.12.1825
Krska J, Cromarty JA, Arris F, et al.	2001	Pharmacist-led medication review in patients over 65: a randomized, controlled trial in primary care	10.1093/ageing/30.3.205
Eakin EG, Bull SS, Riley KM, Reeves MM, McLaughlin P, Gutierrez S.	2007	Resources for health: a primary-care-based diet and physical activity intervention targeting urban Latinos with multiple chronic conditions	10.1037/0278-6133.26.4.392
Hochhalter AK, Song J, Rush J, Sklar L, Stevens A.	2010	Making the Most of Your Healthcare intervention for older adults with multiple chronic illnesses	10.1016/j.pec.2010.01.018
Boult C, Reider L, Leff B, et al.	2011	The effect of guided care teams on the use of health services: results from a cluster-randomized controlled trial	10.1001/archinternmed.2010.540
Garvey J, Connolly D, Boland F, Smith SM.	2015	OPTIMAL, an occupational therapy led self-management support programme for people with multimorbidity in primary care: a randomized controlled trial	10.1186/s12875-015-0267-0
Köberlein-Neu J, Mennemann H, Hamacher S, et al.	2016	Interprofessional Medication Management in Patients With Multiple Morbidities	10.3238/arztebl.2016.0741
Mercer, S.W., Fitzpatrick, B., Guthrie, B. *et al.*	2016	The CARE Plus study – a whole-system intervention to improve quality of life of primary care patients with multimorbidity in areas of high socioeconomic deprivation: exploratory cluster randomised controlled trial and cost-utility analysis	10.1186/s12916-016-0634-2
González-Ortega M, Gené-Badia J, Kostov B, García-Valdecasas V, Pérez-Martín C.	2017	Randomized trial to reduce emergency visits or hospital admissions using telephone coaching to complex patients	10.1093/fampra/cmw119
Jäger C, Freund T, Steinhäuser J, et al.	2017	Impact of a tailored program on the implementation of evidence-based recommendations for multimorbid patients with polypharmacy in primary care practices-results of a cluster-randomized controlled trial	10.1186/s13012-016-0535-y
Muth C, Uhlmann L, Haefeli WE, et al.	2018	Effectiveness of a complex intervention on Prioritising Multimedication in Multimorbidity (PRIMUM) in primary care: results of a pragmatic cluster randomised controlled trial	10.1136/bmjopen-2017-017740

Reed RL, Roeger L, Howard S, et al.	2018	A self-management support program for older Australians with multiple chronic conditions: a randomised controlled trial	10.5694/mja17.00127
Salisbury C, Man MS, Bower P, et al.	2018	Management of multimorbidity using a patient-centred care model: a pragmatic cluster-randomised trial of the 3D approach	10.1016/S0140-6736(18)31308-4
Schäfer I, Kaduszkiewicz H, Mellert C, et al.	2018	Narrative medicine-based intervention in primary care to reduce polypharmacy: results from the cluster-randomised controlled trial MultiCare AGENDA	10.1136/bmjopen-2017-017653
Contant É, Loignon C, Bouhali T, Almirall J, Fortin M.	2019	A multidisciplinary self-management intervention among patients with multimorbidity and the impact of socioeconomic factors on results	10.1186/s12875-019-0943-6

Intention-to-treat	Unique ID	Study ID	Experimental	Comparator	Outcome	Weight	D1	D2	D3	D4	D5	Overall
	101_A	Chan 2023	SUMMIT	EUC	Hospitalisation Rate	1	+	+	+	+	+	+
	101_B	Chan 2023	SUMMIT	EUC	ED Visit Rate	1	+	+	+	+	+	+
	101_C	Chan 2023	SUMMIT	EUC	HRQoL (SF-12) Physical	1	+	+	+	!	+	!
	101_D	Chan 2023	SUMMIT	EUC	HRQoL (SF-12) Mental	1	+	+	+	!	+	!
	101_E	Chan 2023	SUMMIT	EUC	Primary Care Visit	1	+	+	+	+	+	+
	101_F	Chan 2023	SUMMIT	EUC	Behavioural health visit	1	+	+	+	+	+	+
	102_A	Fisher 2020	Multimorbidity Intervention	Usual Home Care	HRQoL (SF-12) Physical	1	+	+	+	+	+	+
	102_B	Fisher 2020	Multimorbidity Intervention	Usual Home Care	HRQoL (SF-12) Mental	1	+	+	+	+	+	+
	102_C	Fisher 2020	Multimorbidity Intervention	Usual Home Care	HSSUI Total Cost	1	+	+	+	+	+	+
	102_D	Fisher 2020	Multimorbidity Intervention	Usual Home Care	No. of participants with hospitalisation	1	+	+	+	+	!	!
	102_E	Fisher 2020	Multimorbidity Intervention	Usual Home Care	No. of hospitalisation	1	+	+	+	+	!	!
	102_F	Fisher 2020	Multimorbidity Intervention	Usual Home Care	No. of participants with ED Visits	1	+	+	+	+	!	!
	102_G	Fisher 2020	Multimorbidity Intervention	Usual Home Care	No. of ED Visits	1	+	+	+	+	+	+
	102_H	Fisher 2020	Multimorbidity Intervention	Usual Home Care	Prescription Meds	1	+	+	+	+	+	+
	102_I	Fisher 2020	Multimorbidity Intervention	Usual Home Care	Ambulance & 911	1	+	+	+	+	+	+
	102_J	Fisher 2020	Multimorbidity Intervention	Usual Home Care	Emergency Department	1	+	+	+	+	+	+
	102_K	Fisher 2020	Multimorbidity Intervention	Usual Home Care	Family Physician	1	+	+	+	+	+	+
	102_L	Fisher 2020	Multimorbidity Intervention	Usual Home Care	Hospital	1	+	+	+	+	+	+
	102_M	Fisher 2020	Multimorbidity Intervention	Usual Home Care	Physician Specialist	1	+	+	+	+	+	+
	103_A	Fortin 2021	Interdisciplinary Multifaceted Interventic	Usual Care	HRQoL (EQ-5D-5L)	1	+	+	!	!	!	!
	103_B	Fortin 2021	Interdisciplinary Multifaceted Interventic	Usual Care	VR-12 Physical component summary	1	+	+	!	!	!	!
	103_C	Fortin 2021	Interdisciplinary Multifaceted Interventic	Usual Care	VR-12 Mental component summary	1	+	+	!	!	!	!
	104_A	Gillespie 2022	OPTIMAL Programme	Usual Primary Care	HRQoL (EQ-5D-3L), 3 months	1	+	+	+	!	+	!
	104_B	Gillespie 2022	OPTIMAL Programme	Usual Primary Care	HRQoL (EQ-5D-3L), 6 months	1	+	+	+	!	+	!
	104_C	Gillespie 2022	OPTIMAL Programme	Usual Primary Care	GP Visits	1	+	+	+	!	+	!
	104_D	Gillespie 2022	OPTIMAL Programme	Usual Primary Care	Outpatient Visits	1	+	+	+	!	+	!
	104_E	Gillespie 2022	OPTIMAL Programme	Usual Primary Care	Outpatient Visits	1	+	+	+	!	+	!
	104_F	Gillespie 2022	OPTIMAL Programme	Usual Primary Care	A&E Visits	1	+	+	+	!	+	!
	104_G	Gillespie 2022	OPTIMAL Programme	Usual Primary Care	A&E visits (Cost)	1	+	+	+	+	+	+
	104_H	Gillespie 2022	OPTIMAL Programme	Usual Primary Care	GP Visits (Cost)	1	+	+	+	+	+	+
	104_I	Gillespie 2022	OPTIMAL Programme	Usual Primary Care	Inpatient nights (Cost)	1	+	+	+	+	+	+
	104_J	Gillespie 2022	OPTIMAL Programme	Usual Primary Care	Outpatient Visits (Cost)	1	+	+	+	+	+	+
	104_K	Gillespie 2022	OPTIMAL Programme	Usual Primary Care	Total cost	1	+	+	+	+	+	+
	105_A	Khurti 2021	MAP Programme	Usual Disease Management	HRQoL (EQ-5D-5L)	1	+	+	+	!	+	!
	105_B	Khurti 2021	MAP Programme	Usual Disease Management	HRQoL (EQ-VAS)	1	+	+	+	!	+	!
	106_A	Lear 2021	Internet CDM Program	Usual Care	All-cause Hospitalisation	1	+	+	+	+	+	+
	106_B	Lear 2021	Internet CDM Intervention	Usual Care	All-cause In-patient Days	1	+	+	+	+	+	+
	106_C	Lear 2021	Internet CDM Program	Usual Care	Unique (at least 1) all-cause hospitalisation	1	+	+	+	+	-	-
	106_D	Lear 2021	Internet CDM Program	Usual Care	Composite All-cause Hospitalisation or Death	1	+	+	+	+	-	-
	106_E	Lear 2021	Internet CDM Program	Usual Care	Time to First Hospitalisation	1	+	+	+	+	-	-
	106_F	Lear 2021	Internet CDM Program	Usual Care	HRQoL (SF-36), Physical Functioning	1	+	+	+	!	+	!
	106_G	Lear 2021	Internet CDM Program	Usual Care	HRQoL (SF-36), General health	1	+	+	+	!	+	!
	107_A	Lee 2021	Integrated Multidomain Intervention	Usual Care	HRQoL (SF-36) Physical, 3 months	1	+	-	+	!	+	-
	107_B	Lee 2021	Integrated Multidomain Intervention	Usual Care	HRQoL (SF-36) Physical, 6 months	1	+	-	!	!	+	-
	107_C	Lee 2021	Integrated Multidomain Intervention	Usual Care	HRQoL (SF-36) Physical, 9 months	1	+	-	!	!	+	-
	107_D	Lee 2021	Integrated Multidomain Intervention	Usual Care	HRQoL (SF-36) Physical, 12 months	1	+	-	!	!	+	-
	107_E	Lee 2021	Integrated Multidomain Intervention	Usual Care	HRQoL (SF-36) Physical, Overall	1	+	-	!	!	+	-
	107_F	Lee 2021	Integrated Multidomain Intervention	Usual Care	HRQoL (SF-36) Mental, 3 months	1	+	-	+	!	+	-
	107_G	Lee 2021	Integrated Multidomain Intervention	Usual Care	HRQoL (SF-36) Mental, 6 months	1	+	-	!	!	+	-
	107_H	Lee 2021	Integrated Multidomain Intervention	Usual Care	HRQoL (SF-36) Mental, 9 months	1	+	-	!	!	+	-
	107_I	Lee 2021	Integrated Multidomain Intervention	Usual Care	HRQoL (SF-36) Mental, 12 months	1	+	-	!	!	+	-
	107_J	Lee 2021	Integrated Multidomain Intervention	Usual Care	HRQoL (SF-36) Mental, Overall	1	+	-	!	!	+	-
	108_A	O'Toole 2021	OPTIMAL Programme	Usual Care	HRQoL (EQ-VAS), at immediate f/up	1	+	+	+	!	+	!
	108_B	O'Toole 2021	OPTIMAL Programme	Usual Care	HRQoL (EQ-5D-3L), at immediate f/up	1	+	+	+	!	+	!
	108_C	O'Toole 2021	OPTIMAL Programme	Usual Care	HRQoL (EQ-VAS), 6-month f/up	1	+	+	+	!	+	!
	108_D	O'Toole 2021	OPTIMAL Programme	Usual Care	HRQoL (EQ-5D-3L), 6-month f/up	1	+	+	+	!	+	!
	108_E	O'Toole 2021	OPTIMAL Programme	Usual Care	GP Visits	1	+	+	+	!	+	!
	108_F	O'Toole 2021	OPTIMAL Programme	Usual Care	Emergency visits	1	+	+	+	!	+	!
	108_G	O'Toole 2021	OPTIMAL Programme	Usual Care	Hospital outpatients	1	+	+	+	!	+	!
	108_H	O'Toole 2021	OPTIMAL Programme	Usual Care	Hospital visits	1	+	+	+	!	+	!
	108_I	O'Toole 2021	OPTIMAL Programme	Usual Care	Hospital nights	1	+	+	+	!	+	!
	109_A	Shang-Lin 2020	Home based telehealth exercise training	Usual lifestyles and routine outp	HRQoL (SF-36) Physical	1	+	+	+	!	+	!
	109_B	Shang-Lin 2020	Home based telehealth exercise training	Usual lifestyles and routine outp	HRQoL (SF-36) Mental	1	+	+	+	!	+	!
	110_A	Stewart 2021	TIP Programme	Usual Care	HRQoL (EQ-5D)	1	+	+	+	!	+	!
	110_B	Stewart 2021	TIP Programme	Usual Care	Health Status (VR-12), Physical	1	+	+	+	!	+	!
	110_C	Stewart 2021	TIP Programme	Usual Care	Health Status (VR-12), Mental	1	+	+	+	!	+	!
	111_A	Yang 2022	Nurse-led Medication Self-management	Usual Care	HRQoL (EQ-5D-5L), time 1	1	+	+	!	!	+	!
	111_B	Yang 2022	Nurse-led Medication Self-management	Usual Care	HRQoL (EQ-5D-5L), time 2	1	+	+	-	!	+	-
	111_C	Yang 2022	Nurse-led Medication Self-management	Usual Care	HRQoL (EQ-VAS), time 1	1	+	+	!	!	+	!
	111_D	Yang 2022	Nurse-led Medication Self-management	Usual Care	HRQoL (EQ-VAS), time 2	1	+	+	-	!	+	-
	111_E	Yang 2022	Nurse-led Medication Self-management	Usual Care	CHC visits, time 2	1	+	+	-	+	+	-
	111_F	Yang 2022	Nurse-led Medication Self-management	Usual Care	Outpatient consultations, time 2	1	+	+	-	+	+	-
	111_G	Yang 2022	Nurse-led Medication Self-management	Usual Care	ED visits, time 2	1	+	+	-	+	+	-
	111_H	Yang 2022	Nurse-led Medication Self-management	Usual Care	Hospital admission, time 2	1	+	+	-	+	+	-
	111_I	Yang 2022	Nurse-led Medication Self-management	Usual Care	Days in hospital, time 2	1	+	+	-	+	+	-
	111_J	Yang 2022	Nurse-led Medication Self-management	Usual Care	Medical costs, time 2	1	+	+	-	+	+	-
	401_A	Garvey 2015	OPTIMAL	Usual Care	HRQoL (EQ-VAS)	1	+	+	+	!	+	!
	401_B	Garvey 2015	OPTIMAL	Usual Care	GP Visits	1	+	+	+	+	+	+
	401_C	Garvey 2015	OPTIMAL	Usual Care	PN Visits	1	+	+	+	+	+	+
	401_D	Garvey 2015	OPTIMAL	Usual Care	Hosp Admissions	1	+	+	+	+	+	+
	402_A	Gonzales Ortega 2017	Telephone Coaching Intervention	Usual Care	Total urgent visits (24hES + UCC + ER + UHA)	1	+	!	+	+	+	!
	402_B	Gonzales Ortega 2017	Telephone coaching intervention	Usual care	Elective hospital admission	1	+	!	+	+	+	!
	402_C	Gonzales Ortega 2017	Telephone coaching intervention	Usual Care	Elective hospital admission, days	1	+	!	+	+	+	!
	402_D	Gonzales Ortega 2017	Telephone coaching intervention	NA	HRQoL (SF-12), PCS	1	+	!	+	!	+	!
	402_E	Gonzales Ortega 2017	Telephone coaching intervention	Usual Care	HRQoL (SF-12), MCS	1	+	!	+	!	+	!
	403_A	Reed 2018	CDSMS Programme	Positive attention	GP visits	1	+	+	+	+	+	+
	403_B	Reed 2018	CDSMS Programme	Attention Programme	ED visits	1	+	+	+	+	+	+
	403_C	Reed 2018	CDSMS Programme	Attention Programme	Admissions	1	+	+	+	+	+	+
	601_A	Kiely 2024	LinkMM Intervention	Wait-list Usual Care	HRQoL (EQ-5D-5L)	1	+	+	+	!	+	!
	601_B	Kiely 2024	LinkMM Intervention	Wait-list Usual Care	HRQoL (EQ-VAS)	1	+	+	+	!	+	!
	601_C	Kiely 2024	LinkMM Intervention	Wait-list Usual Care	GP visits	1	+	+	+	+	+	+
	601_D	Kiely 2024	LinkMM Intervention	Wait-list Usual Care	GP Nurse visits	1	+	+	+	+	+	+
	601_E	Kiely 2024	LinkMM Intervention	Wait-list Usual Care	OOH	1	+	+	+	+	+	+
	601_F	Kiely 2024	LinkMM Intervention	Wait-list Usual Care	A&E	1	+	+	+	+	+	+
	601_G	Kiely 2024	LinkMM Intervention	Wait-list Usual Care	LOHS (days)	1	+	+	+	+	+	+
	601_H	Kiely 2024	LinkMM Intervention	Wait-list Usual Care	OPD	1	+	+	+	+	+	+
	601_I	Kiely 2024	LinkMM Intervention	Wait-list Usual Care	Total cost	1	+	+	+	+	+	+
	602_A	Tew 2024	Gentle Years Yoga Programme	Usual Care	HRQoL (EQ-5D-5L)	1	+	+	+	!	+	!
	602_B	Tew 2024	Gentle Years Yoga Programme	Usual Care	HRQoL (EQ-VAS)	1	+	+	+	!	+	!
	602_C	Tew 2024	Gentle Years Yoga Programme	Usual Care	PROMIS-29, Physical	1	+	+	+	!	+	!
	602_D	Tew 2024	Gentle Years Yoga Programme	Usual Care	PROMIS-29, Mental	1	+	+	+	!	+	!
	602_E	Tew 2024	Gentle Years Yoga Programme	Usual Care	GP visits	1	+	+	+	+	+	+
	602_F	Tew 2024	Gentle Years Yoga Programme	Usual Care	GP Nurse visits	1	+	+	+	+	+	+
	602_G	Tew 2024	Gentle Years Yoga Programme	Usual Care	Physio visits (Community)	1	+	+	+	+	+	+
	602_H	Tew 2024	Gentle Years Yoga Programme	Usual Care	OPD	1	+	+	+	+	+	+
	602_I	Tew 2024	Gentle Years Yoga Programme	Usual Care	A&E	1	+	+	+	+	+	+
	602_J	Tew 2024	Gentle Years Yoga Programme	Usual Care	Inpatient nights	1	+	+	+	+	+	+
	602_K	Tew 2024	Gentle Years Yoga Programme	Usual Care	GP visits cost	1	+	+	+	+	+	+
	602_L	Tew 2024	Gentle Years Yoga Programme	Usual Care	GP Nurse visits cost	1	+	+	+	+	+	+
	602_M	Tew 2024	Gentle Years Yoga Programme	Usual Care	Physio visits (Community) cost	1	+	+	+	+	+	+
	602_N	Tew 2024	Gentle Years Yoga Programme	Usual Care	OPD cost	1	+	+	+	+	+	+
	602_O	Tew 2024	Gentle Years Yoga Programme	Usual Care	Inpatient nights cost	1	+	+	+	+	+	+
	602_P	Tew 2024	Gentle Years Yoga Programme	Usual Care	A&E cost	1	+	+	+	+	+	+
	602_Q	Tew 2024	Gentle Years Yoga Programme	Usual Care	Medication cost	1	+	+	+	+	+	+
	602_R	Tew 2024	Gentle Years Yoga Programme	Usual Care	Total cost (incl intervention)	1	+	+	+	+	+	+

+ Low risk
! Some concerns
- High risk

D1 Randomisation process
D2 Deviations from the intended interventions
D3 Missing outcome data
D4 Measurement of the outcome
D5 Selection of the reported result

Fig. 1: Summary of Risk of Bias for Each Outcome for Randomised Trials

Intention-to-treat	Unique ID	Study ID	Experimental	Comparator	Outcome	Weight	D1a	D1b	D2	D3	D4	D5	Overall
	201_A	Kinchin 2022	OPEN ARCH	Usual Care	HRQoL (AQoL-8D)	1	+	+	+	!	!	+	!
	201_B	Kinchin 2022	OPEN ARCH	Usual Care	HRQoL (EQ-5D-3L)	1	+	+	+	!	!	!	!
	201_C	Kinchin 2022	OPEN ARCH	Usual Care	Inpatient	1	+	+	+	!	+	+	!
	201_D	Kinchin 2022	OPEN ARCH	Usual Care	ALOS	1	+	+	+	!	+	+	!
	201_E	Kinchin 2022	OPEN ARCH	Usual Care	Ambulance	1	+	+	+	!	+	+	!
	201_F	Kinchin 2022	OPEN ARCH	Usual Care	ED	1	+	+	+	!	+	+	!
	201_G	Kinchin 2022	OPEN ARCH	Usual Care	Allied Health Service	1	+	+	+	!	+	+	!
	201_H	Kinchin 2022	OPEN ARCH	Usual Care	Home/social support service	1	+	+	+	!	+	+	!
	201_I	Kinchin 2022	OPEN ARCH	Usual Care	Inpatient Cost	1	+	+	+	+	+	+	+
	201_J	Kinchin 2022	OPEN ARCH	Usual Care	Ambulance Cost	1	+	+	+	+	+	+	+
	201_K	Kinchin 2022	OPEN ARCH	Usual Care	ED Cost	1	+	+	+	+	+	+	+
	201_L	Kinchin 2022	OPEN ARCH	Usual Care	Allied Health Service Cost	1	+	+	+	+	+	+	+
	201_M	Kinchin 2022	OPEN ARCH	Usual Care	Home/social support service cost	1	+	+	+	+	+	+	+
	201_N	Kinchin 2022	OPEN ARCH	Usual Care	Patient OOP	1	+	+	+	+	+	+	+
	201_O	Kinchin 2022	OPEN ARCH	Usual Care	Cost (Total HS including Intervention)	1	+	+	+	+	+	+	+
	202_A	Sturm 2022	HoPES3	Usual Care	HRQoL (SF-12) Physical	1	+	-	+	!	!	+	-
	202_B	Sturm 2022	HoPES3	Usual Care	HRQoL (SF-12) Mental	1	+	-	+	+	!	+	-
	501_A	Boult 2011	Guided Care (GC)	Usual Care	Hoospital admissions	1	+	+	+	+	+	+	+
	501_B	Boult 2011	Guided Care (GC)	Usual Care	Hospital 30-day readmission	1	+	+	+	+	+	+	+
	501_C	Boult 2011	Guided Care (GC)	Usual Care	Hospital days	1	+	+	+	+	+	+	+
	501_D	Boult 2011	Guided Care (GC)	Usual Care	Skilled nursing facility admissions	1	+	+	+	+	+	+	+
	501_E	Boult 2011	Guided Care (GC)	Usual Care	Skilled nursing facility days	1	+	+	+	+	+	+	+
	501_F	Boult 2011	Guided Care (GC)	Usual Care	Emergency department visits	1	+	+	+	+	+	+	+
	501_G	Boult 2011	Guided Care (GC)	Usual Care	Primary care visits	1	+	+	+	+	+	+	+
	501_H	Boult 2011	Guided Care (GC)	Usual Care	Specialist visits	1	+	+	+	+	+	+	+
	501_I	Boult 2011	Guided Care (GC)	Usual Care	Home health care episodes	1	+	+	+	+	+	+	+
	502_A	Koberlein Neu 2016	WESTGEM intervention	Usual Care	HRQoL (SF-12), PCS	1	+	+	+	+	!	+	!
	502_B	Koberlein Neu 2016	WESTGEM Intervention	Usual Care	HRQoL (SF-12), MCS	1	+	+	+	+	!	+	!
	503_A	Mercer 2016	CARE Plus	Usual Care	HRQoL (EQ-5D-5L), 6 months	1	+	+	+	+	+	+	+
	503_B	Mercer 2016	CARE Plus	Usual Care	HRQoL (EQ-5D-5L), 12 months	1	+	+	+	+	+	+	+
	503_C	Mercer 2016	CARE Plus	Usual Care	HRQoL (W-BQ12) General Well-being, 6 months	1	+	+	+	+	+	+	+
	503_D	Mercer 2016	CARE Plus	Usual Care	HRQoL (W-BQ12) General Well-being, 12 months	1	+	+	+	+	+	+	+
	504_A	Muth 2018	PRIMIUM	Usual Care	HRQoL (EQ-5D), 6 months	1	+	+	+	+	!	+	!
	504_B	Muth 2018	PRIMIUM	Usual Care	HRQoL (EQ-5D), 9 months	1	+	+	+	+	!	+	!
	504_C	Muth 2018	PRIMIUM	Usual Care	Number of hospital stays, 6 months	1	+	+	+	+	+	+	+
	504_D	Muth 2018	PRIMIUM	Usual Care	Number of hospital stays, 9 months	1	+	+	+	+	+	+	+
	504_E	Muth 2018	PRIMIUM	Usual Care	Number of days spent in hospital, 6 months	1	+	+	+	+	+	+	+
	504_F	Muth 2018	PRIMIUM	Usual Care	Number of days spent in hospital, 9 months	1	+	+	+	+	+	+	+
	505_A	Salisbury 2018	3D Intervention	Usual Care	HRQoL (EQ-5D-5L)	1	+	+	+	+	!	+	!
	505_B	Salisbury 2018	3D Intervention	Usual Care	Primary care physician consultations	1	+	+	+	+	+	+	+
	505_C	Salisbury 2018	3D Intervention	Usual Care	Nurse consultations	1	+	+	+	+	+	+	+
	505_D	Salisbury 2018	3D Intervention	Usual Care	Hospital admissions	1	+	+	+	+	+	+	+
	505_E	Salisbury 2018	3D Intervention	Usual Care	Hospital outpatient attendances	1	+	+	+	+	+	+	+
	506_A	Schafer 2018	MultiCare	Usual Care	EQ-5D	1	+	+	+	+	!	+	!
	506_B	Schafer 2018	MultiCare	Usual Care	Contacts with GPs	1	+	+	+	+	+	+	+
	506_C	Schafer 2018	MultiCare	Usual Care	Contacts with other outpatient physicians	1	+	+	+	+	+	+	+
	506_D	Schafer 2018	MultiCare	Usual Care	Physical, occupational or speech therapy units	1	+	+	+	+	+	+	+
	506_E	Schafer 2018	MultiCare	Usual Care	Days spent in hospital	1	+	+	+	+	+	+	+
	507_A	Sommers 2000	SCC Intervention	Usual Care	Hospital admissions	1	+	+	+	+	+	!	!
	507_B	Sommers 2000	SCC Intervention	Usual Care	≥ 1 60-d readmissions	1	+	+	+	+	+	!	!
	507_C	Sommers 2000	SCC Intervention	Usual Care	No. of participants with hospitalisation	1	+	+	+	+	+	!	!
	507_D	Sommers 2000	SCC Intervention	Usual Care	Other physician visits	1	+	+	+	+	+	!	!
	507_E	Sommers 2000	SCC Intervention	Usual Care	HRQoL (SF-36)	1	+	+	+	-	!	!	-
	203_A	McCarthy 2022	SPPiRE Intervention	Usual Care	HRQoL (ED-5D-5L)	1	+	+	+	+	!	+	!
	203_B	McCarthy 2022	SPPiRE Intervention	Usual Care	GP visits	1	+	+	+	+	+	+	+
	203_C	McCarthy 2022	SPPiRE Intervention	Usual Care	Emergency visits	1	+	+	+	+	+	+	+
	203_D	McCarthy 2022	SPPiRE Intervention	Usual care	Hospital nights	1	+	+	+	+	+	+	+
	203_E	McCarthy 2022	SPPiRE Intervention	Usual Care	Hospital outpatients	1	+	+	+	+	+	+	+
	204_A	Gillespie 2025	SPPiRE Intervention	Usual Care	GP Visits (Cost)	1	+	+	+	+	+	+	+
	204_B	Gillespie 2025	SPPiRE Intervention	Usual Care	Outpatient Visits (Cost)	1	+	+	+	+	+	+	+
	204_C	Gillespie 2025	SPPiRE Intervention	Usual Care	Inpatient nights (Cost)	1	+	+	+	+	+	+	+
	204_D	Gillespie 2025	SPPiRE Intervention	Usual Care	A&E visits (Cost)	1	+	+	+	+	+	+	+
	204_E	Gillespie 2025	SPPiRE Intervention	Usual Care	Nurse visits (Cost)	1	+	+	+	+	+	+	+
	204_F	Gillespie 2025	SPPiRE Intervention	Usual Care	Total cost	1	+	+	+	+	+	+	+

+ Low risk
! Some concerns
- High risk

D1a Randomisation process
D1b Timing of identification or recruitment of participants
D2 Deviations from the intended interventions
D3 Missing outcome data
D4 Measurement of the outcome
D5 Selection of the reported result

Fig. 2: Summary of Risk of Bias for Each Outcome for Cluster-randomised Trials

ROBINS-I

Unique ID	Study ID	Experimental	Comparator	Outcome	D1	D2	D3	D4	D5	D6	D7	Overall		
301_A	Hong 2021	QIP Intervention	Historical Control	Proportion of QIP Patients with Healthcare Resource Utilisation	x	+	+	+	-	+	+	x	+	Low
301_B	Hong 2021	QIP Intervention	Concurrent Control	Proportion of QIP Patients with Healthcare Resource Utilisation	x	+	+	+	-	+	+	x	-	Moderate
301_C	Hong 2021	QIP Intervention	Historical Control	Average number of healthcare visits	x	+	+	+	-	+	+	x	x	Serious
301_D	Hong 2021	QIP Intervention	Concurrent Control	Average number of healthcare visits	x	+	+	+	-	+	+	x	!	Critical
301_E	Hong 2021	QIP Intervention	Historical Control	Medication Utilisation	x	+	+	+	-	+	+	x		
301_F	Hong 2021	QIP Intervention	Concurrent Control	Medication Utilisation	x	+	+	+	-	+	+	x	D1	Bias due to confounding
302_A	Mateo-Abad 2020a	CareWell Program	Usual Care	Hospitalisation	-	+	+	+	-	+	-	-	D2	Bias in selection of participants into the study
302_B	Mateo-Abad 2020a	CareWell Program	Usual Care	(re-)Hospitalisation (among hospitalized)	-	+	+	+	-	+	-	-	D3	Bias in classification of interventions
302_C	Mateo-Abad 2020a	CareWell Program	Usual Care	Days in hospital (among hospitalized)	-	+	+	+	-	+	-	-	D4	Bias due to deviations from intended interventions
302_D	Mateo-Abad 2020a	CareWell Program	Usual Care	Visits to ER	-	+	+	+	-	+	-	-	D5	Bias due to missing data
302_E	Mateo-Abad 2020a	CareWell Program	Usual Care	Contacts with GP+	-	+	+	+	-	+	-	-	D6	Bias in measurement of outcomes
302_F	Mateo-Abad 2020a	CareWell Program	Usual Care	Contacts with GP Nurse	-	+	+	+	-	+	-	-	D7	Bias in selection of the reported result

Fig. 3: Summary of Risk of Bias for Each Outcome for Non-randomised Trials

Tab. 1 Characteristics of the included studies

Study ID, Study Design, Study Location (Country)	Settings and Funding Sources	Patients' Characteristics; Duration of Intervention and Follow-up period	Multimorbidity Operational Definition	TIDieR Checklist: Brief name of the intervention; Why: Aim of the study; What: Procedure and Materials; Who provided the intervention; How, Where, When and How much, and Comparison	Primary Outcome
**Medicine management**					
Yang 2022<br>RCT<br>Hong Kong, China	Community Care; No specific grant from funding agencies in the public, commercial or not-for-profit sectors	136 patients; 60.2% female; mean age 71.13; mean 4.73 conditions; intervention 6 weeks; follow-up at 6 weeks (immediately post-intervention), and 3 months	≥ 60 years, having at least 3 of the 38 chronic conditions listed in the trial protocol	Intervention: Nurse-led Medication Self-management;<br>Aim: To evaluate the effectiveness of a nurse-led medication self-management intervention on medication adherence and health outcomes in older people with multimorbidity;<br>The intervention consisted of three one-on-one educational sessions on medication-related information, motivation and self-management skills and two follow-up phone calls;<br>Providers: Nurse;<br>Face-to-face; phone call (1:1); Three community health centres;<br>Comparison: Usual Care	Medication adherence
Jager 2017<br>CRT<br>Germany	Primary Care; Grant from European Union Framework	273 patients; 55.7% female; mean age 72.2; mean 5.7 conditions;<br>Intervention 9 months; follow-up at intervention completion	>50 years, with at least 3 chronic diseases, more than 4 drugs, and at high risk for medication-related events	Intervention: PomP: A tailored medicines management programme;<br>Aim: To assess the effect of a tailored programme to improve the implementation of three important processes of care for this patient group: (a) structured medication counselling including brown bag reviews, (b) the use of medication lists, and (c) structured medication reviews to reduce potentially inappropriate medication;<br>Training and resources for general practitioners (GPs) and medical	Summary score of 10 prescribing indicators

				assistants: 4-h workshop Patients: educational materials, electronic information tool and reminders for patients. Implementation action plans for each GP practice with focus on three priority actions for medicines management and consideration of patient preferences;<br>Providers: GP, with mean 4.6 medical assistants per practice;<br>In-person: 22 GPs from 18 practices of 66 GP Quality Circles;<br>Comparison: Usual care plus GPs informed of prescribing targets and aware of which patients identified for the trial as high risk	
Koberlein Neu 2016<br>CRT<br>Germany	Primary Care; Funding from the Ziel-2-Förderreihe IuK & Gender med.NRW from the federal state of North Rhine Westphalia and the European Union	162 patients; 53.3% female; mean age 76.8; mean 12.7 conditions;<br>Intervention 15 months, variable intervention exposure based on stepped wedge design. Data extracted for first phase of 3 months when was intervention vs control and no variation in exposure	≥ 65 years, with ≥ 3 chronic disorders affecting two different organ systems, at least one cardiovascular disease, at least one visit to the PCP in each of the preceding three-month intervals, five or more long-term medicines	Intervention: WESTGEM intervention;<br>Aim: To evaluate the effectiveness of interprofessional medication management for elderly multimorbid patients;<br>Comprehensive medication management. Medication management with primary care physicians (PCPs) who sent e-information to home care specialists. Care provided by home-care specialists using case management, conducting a home visit and assessment, and communicating this to a pharmacist who undertook a medicines review and made recommendations. PCPs then responsible for delivering recommendations;<br>Providers: 12 PCPs and attached home care specialists, pharmacist (number not reported);	Quality of medication therapy (MAI score)

				In-person; General practices from two regions in the Westphalia-Lippe area;<br>Comparison: Usual care with their PCP	
Krska 2001<br>RCT<br>UK	Primary Care; Grampian Healthcare NHS Trust	332 patients; 56.5% female; mean age 75; mean 3.9 conditions; Intervention 3 months; follow-up 3 months after drug review	≥ 65 with ≥ 2 conditions and on ≥ 4 medicines	Intervention: Pharmacist-led medication reviews;<br>Aim: To evaluate the effects of pharmacist-led medication reviews in elderly patients taking multiple medications;<br>To evaluate the effects of pharmacist-led medication reviews in elderly patients taking multiple medications;<br>Providers: Clinical pharmacist, General Practitioners (numbers not reported);<br>In-person; Grampian medical practices;<br>Comparison: Usual care and had review of drug therapy by pharmacist but no pharmaceutical care plan implemented	Pharmaceutical care issues
Muth 2018<br>CRT<br>Germany	Primary Care; Grant from the German Federal Ministry of Education and Research	505 patients; 52% female; mean age 72; Intervention delivered over two sessions (HCA and then GP) sessions, lasting 35-45 min each; Follow-up at 6 and 9 months	≥ 60 years, ≥ 3 chronic conditions, ≥ 5 long-term medicines	Intervention: PRIMUM: Prioritising Multi-medication in Multimorbidity;<br>Aim: To improve the appropriateness of medication in older patients with multimorbidity in general practice;<br>Pre-intervention training of 90-120 min for healthcare assistant (HCA) and GP. HCA conducted a checklist-based interview with patients on medication-related problems and a brown bag review to reconcile their medications. HCA entered details into the computerised decision support system (CDSS) GP undertook a review assisted by the CDSS and optimised medication,	Medication Appropriateness Index (MAI)

				discussed it with patients and adjusted it accordingly;<br>Providers: 72 general practices and had to have HCA with access to internet;<br>In-person; 72 general practices;<br>Comparison: Usual care but the control practice teams also received the GP guidelines for ambulatory geriatric care to harmonise usual care in both groups	
McCarthy 2022*<br>CRT<br>Ireland	Primary Care; Research Grant	404 patients; 57% female; mean age 76.5; Intervention 6 months; Follow-up 6 months	⩾ 65 years, with complex multimorbidity, defined as being prescribed ⩾ 15 regular medicines	Intervention: Supporting Prescribing in Older Adults with Multimorbidity in Irish Primary Care (SPPiRE) intervention;<br>Aim: To investigate the effect of a general practitioner (GP)-delivered, individualised medication review in reducing polypharmacy and potentially inappropriate prescriptions (PIPs) in community-dwelling older patients with multimorbidity in primary care;<br>The SPPiRE medication review process had 2 main components: gather and record information and then to discuss and agree upon any changes with their patient based on the recorded information, with a focus on deprescribing medicines that were potentially inappropriate;<br>Providers: GP;<br>In-person; GP practices;<br>Comparison: Usual care	Number of repeat medicines, and the proportion of patients with any potentially inappropriate prescribing (PIP)
Gillespie 2025*<br>Economic evaluation alongside CRT<br>Ireland	Primary Care; Research Grant	403 patients; 57% female; mean age 76.5; Intervention 6 months; Follow-up 6 months	⩾ 65 years, with complex multimorbidity, defined as being prescribed ⩾ 15 regular medicines	Aim: To investigate the cost efectiveness of a general practitioner (GP) delivered, individualised medication review to reduce polypharmacy and potentially inappropriate prescribing in older	Incremental costs, quality adjusted life years (QALYs)

				patients with multimorbidity in Irish primary care.<br>The SPPiRE medication review process had 2 main components: gather and record information and then to discuss and agree upon any changes with their patient based on the recorded information, with a focus on deprescribing medicines that were potentially inappropriate;<br>Providers: GP;<br>In-person; GP practices;<br>Comparison: Usual care	
**Support for self-management**					
Gillespie 2022*<br>Economic evaluation alongside RCT<br>Ireland	Primary Care or Community Care;<br>Research Grant	149 patients; 69.1% female; mean age 66; mean 4.4 conditions; intervention 6 weeks; follow-up at 6 months	> 40 years, 2 or more chronic conditions, a minimum of 4 repeat medications	Intervention: OPTIMAL Programme;<br>Aim: To examine the cost-effectiveness of a 6-week occupational therapy-led self-management support programme (OPTIMAL) for adults with multimorbidity;<br>The intervention comprised of a group-based programme, facilitated by occupational therapists with input from physiotherapists and pharmacists, who delivered a series of 2.5-h educational and goal-setting sessions with participants over 6 consecutive weeks in primary or community care centres;<br>Providers: Occupational therapists with input from physiotherapist and pharmacist;<br>In-person; Eight Health Service Executive primary care areas; Primary care centres or community resource centres;<br>Comparison: Usual Primary Care	Cost per QALY Gained

Khunti 2021<br>RCT<br>UK	Primary Care; Research Grant	353 patients; 54.39% female; mean age 67.76; mean 4.32 conditions; intervention 12 months; follow-up at 6, and 12 months	40 - 85 years, Coexistence of ≥2 chronic conditions	Intervention: Movement through Active Personalised engagement (MAP) programme;<br>Aim: To investigate the impact of a structured, theoretically driven, self-management group education programme on habitual PA levels in people with multimorbidity;<br>The programme comprised four, 1.5- hour group sessions with person-centred self-monitoring and goal setting, delivered by a trained facilitator at 2-week intervals in local community settings;<br>Providers: Trained facilitator;<br>In-person, motivational text-messages; Local community settings;<br>Comparison: Usual Disease Management	Physical activity
Lear 2021<br>RCT<br>Canada	Primary Care; Research Grant	229 patients; 61.6% male; mean age 70.5; mean 2 conditions; intervention 24 months; follow-up at 24 months	> 19 years, presence of 2 or more of the following 5 conditions: diabetes, heart failure, ischemic heart disease, chronic kidney disease, or chronic obstructive pulmonary disease	Intervention: Internet chronic disease management (CDM) Programme;<br>Aim: To compare the effect of an internet-based self-management and symptom monitoring program targeted to patients with 2 or more chronic diseases (internet chronic disease management [CDM]) with usual care on hospitalizations over a 2-year period;<br>Internet-based self-management program using telephone nursing supports and integration within primary care compared with usual care over a 2-year period;<br>Providers: Nurse, dietitian and an exercise specialist;<br>Telemedicine; 71 primary care clinics in small urban and rural areas;<br>Comparison: Usual Care	Number of all-cause hospitalizations

O'Toole 2021*<br>RCT<br>Ireland	Primary Care; Research Grant	149 patients; 69.1% female; mean age 66; mean 4.4 conditions; intervention 6 weeks; follow-up at 6 months	> 40 years, 2 or more chronic conditions, a minimum of 4 repeat medications	Intervention: OPTIMAL Programme;<br>Aim: To evaluate the effectiveness of a group-based, 6-week, occupational therapy-led self-management support programme (OPTIMAL) for patients with multimorbidity;<br>Group-based programme delivered over 6 consecutive weeks; 2.5 hour session;<br>Providers: Occupational therapists with input from physiotherapist and pharmacist;<br>In-person; Eight Health Service Executive primary care areas; Primary care centres or community resource centres;<br>Comparison: Usual Care	HRQoL (EQ-5D-3L), HRQoL (EQ-VAS); Frequency of activity participation (FAI)
Shang-Lin 2020<br>RCT<br>Taiwan	Community Care; Grants from hospital and Ministry of Science and Technology	50 patients; 72% male; mean age 60; intervention 12 weeks; follow-up at 12 weeks	≥ 18 years, 2 or more (cardiometabolic) conditions (ie, hypertension, type 2 diabetes, hyperlipidaemia, heart disease, metabolic syndrome, or gout)	Intervention: Home based telehealth exercise training programme;<br>Aim: To determine the effectiveness of a 12-week home-based telehealth exercise training programme designed to increase physical activity and exercise capacity and improve health-related quality of life in patients with cardiometabolic multimorbidity;<br>The home-based telehealth exercise training program consisted of 36 individualized home-based exercise training sessions and a weekly reminder for maintenance of exercise and providing patient support;<br>Providers: Medical/rehabilitation physician, nurse, physiotherapist;<br>Initial session in-person and then via telehealth (1:1); Outpatient clinics, telehealth provided at home;<br>Comparison: Usual lifestyles and routine outpatient follow up	Physical activity amount; HRQoL (SF-36)

Sturm 2022<br>CRT<br>Germany	Primary Care; Federal Ministry of Education and Research	297 patients; 54.9% female; mean age 78.46; intervention 6 months; follow-up at 6 months	≥ 70 years, had at least three chronic diseases, were taking at least three medications	Intervention: Holistic Care Programme for Elderly Patients to Integrate Spiritual Needs, Social Activity and Self-Care into Disease Management in Primary Care (HoPES3);<br>Aim: To assess the effectiveness of the HoPES3 intervention at the patient level in terms of primary and secondary outcomes;<br>HoPES3 intervention focuses on three domains: Spiritual needs, Self-care by means of home remedies, Social activity and loneliness;<br>Providers: Primary care physicians (GP), medical assistants;<br>In-person; 24 primary care practices;<br>Comparison: Usual Care	Self-Efficacy for Managing Chronic Disease 6-Item Scale (SES6G)
Eakin 2007<br>RCT<br>USA	Primary Care; Robert Wood Johnson Foundation Grant	200 patients; 79.2% female; mean age 50; intervention 6 months; follow-up at 6 weeks and 6 months	Adults with ≥ 2 conditions (of 14 conditions listed)	Intervention: Self-management support, diet, and exercise intervention based on chronic care model;<br>Aim: To address multiple risk factors in patients targeting low-income, largely Spanish speaking patients with multiple chronic conditions;<br>Patient education materials with three tailored newsletters and linkage to local services. Two structured visits (home or clinic lasting 60-90 min and two follow up telephone contacts over 16 weeks;<br>Providers: An experienced bilingual health educator working in a community health centre providing primary healthcare services to low income and medically underserved individuals;<br>In-person, telemedicine; Home or clinic;	Dietary behaviour and physical activity

				Comparison: Usual care plus a guide to local services and three newsletters	
Garvey 2015<br>RCT<br>Ireland	Primary Care; Health Research Board of Ireland	50 patients; 65.4% female; mean age 66; median 4.5 conditions; intervention 6 weeks; follow-up at 2-week post-intervention follow-up	≥ 2 chronic conditions and 4 repeat medications	Intervention: OPTIMAL, occupational therapy (OT) led self-management support course;<br>Aim: To address the challenges of living with multimorbidity in a primary care setting;<br>Focus on goal setting and prioritisation. Peer support through group meetings. Weekly meetings in local health centre over 6 weeks, meeting duration 2.5 h;<br>Providers: Primary care OTs in each centre led the programme with input from physiotherapist and pharmacist for one session each. Training and intervention manual for OT providers, provided by the research team;<br>In-person; Three primary care centres;<br>Comparison: Wait-list control. Received usual care whilst waiting	Activity participation (Frenchay Activities Index)
Reed 2018<br>RCT<br>Australia	Primary Care; Sharing Health Care Initiative - Innovations in Chronic Disease Self-Management Research Grants programme	254 patients; 59% female; mean age not reported, approximate 50% > 75 years; mean 4.5 conditions;<br>Intervention 6 months with immediate follow-up	> 60 years with ≥ 2 conditions and neutral or poor self-rated health	Intervention: Clinician-led CDSMS Programme;<br>Aim: To determine whether a clinician-led chronic disease self-management support (CDSMS) programme improves the overall self-rated health level of older Australians with multiple chronic health conditions;<br>Clinician-led CDSMS Programme which included goal setting and the development of individualised care plans, based on the Flinders CDSMS programme. Delivered by nurses or psychologists in the patients' home, 3 home visits with 4 follow up phone	Self-rated Health

				calls over 6 months, delivered independently of GP care. Mentoring of clinicians by trained accreditors;<br>Providers: Trained nurses and psychologists, mentor supervising them;<br>In-person, telemedicine; Five general practices;<br>Comparison: Attention control - same number of visits to the study clinicians but did not receive the CDSMS programme	
Tew 2024<br>RCT<br>England and Wales	Primary Care;<br>National Institute for Health and Care Research (NIHR) Health Technology Assessment programme	454 patients; 60.6% female; mean age 73.5; median 3 conditions; intervention 12 weeks; follow-up at 3, 6, and 12 months after intervention	⩾ 65 years, 2 or more of the predefined chronic health condition	Intervention: Gentle Years Yoga (GYY) programme;<br>Aim: To establish if the offer of a 12-week GYY programme in addition to usual care is more effective compared with usual care alone in improving HRQoL (EQ-5D-5L utility index score) over 12 months in people aged 65 years or over with multimorbidity;<br>GYY incorporates physical postures and transitions as well as breathing, concentration and relaxation activity. Each course involved 12, 75-minute sessions of group-based yoga, usually delivered 1 week apart;<br>Providers: Yoga teachers;<br>Face to face or online; 15 general practices;<br>Comparison: Usual Care	EQ-5D-5L utility index score
**Care coordination plus support for self-management**					
Chan 2023<br>RCT<br>USA	Community Care;<br>Academic and Research Grants	159 patients; 65.8% male; mean age 54.9; intervention 6 months; follow-up at 6 months	≥18 years, had 2 or more chronic medical conditions or a chronic condition and a substance use disorder or mental illness	Intervention: SUMMIT A-ICU;<br>Aim: To assess the efficacy of a multidisciplinary ambulatory intensive care unit (A-ICU) intervention on health care utilization and patient-reported outcomes;	Change in hospitalisation rate

				Activities included comprehensive 90-minute intake, transitional care coordination, and flexible appointments, with reduced panel size;<br>Providers: Staffing consisted of 2 physicians (totalling 1 full-time equivalent) with addiction board certification, a complex care nurse, 2 care coordinators, 2 licensed clinical social workers, a pharmacist, a team manager, and a quality analyst;<br>In-person; Health care clinic;<br>Comparison: Enhanced Usual Care	
Fisher 2020<br>RCT<br>Canada	Community Care;<br>Research and MOH Grants	59 patients; 50.8% male; mean 8.6 conditions; intervention 6 months; follow-up at 6 months	65 years of age and older with at least 3 chronic conditions	Intervention: Multimorbidity Intervention;<br>Aim: To determine the effectiveness of a 6-month, community-based, multimorbidity intervention compared to usual home care services for community-dwelling older adults (age 65+ years) with multimorbidity (3+ chronic conditions) that were newly referred to and receiving home care services;<br>The multimorbidity intervention was delivered in addition to usual care, consisted of main components including in-home visits, Monthly case conferences, Case management;<br>Providers: An interprofessional team consisting of a Care Coordinator (CC), Registered Nurse (RN), Physiotherapist (PT), Occupational Therapist (OT), and Personal Support Worker (PSW);<br>In-person; Community Care Access Centre;<br>Comparison: Usual Home Care	HRQoL (SF-12)

Fortin 2021<br>RCT<br>Canada	Primary Care; Research Grant	284 patients; 53.5% female; mean age 61; mean 5 conditions; intervention 4 months; follow-up at 4 months	18 to 80 years with 3 or more chronic conditions	Intervention: Interdisciplinary Multifaceted Intervention;<br>Aim: To measure the effectiveness of a 4-month interdisciplinary multifaceted intervention based on a change in care delivery for patients with multimorbidity in primary care practices;<br>Patient-centred intervention based on a motivational approach and self-management support;<br>Providers: Primary care physicians, nurses, cardiologists, internal medicine specialists, endocrinologists, pneumologists, nutritionists, kinesiologists;<br>In-person; Primary care centre;<br>Comparison: Usual Care	Health Education Impact Questionnaire (heiQ) and Self-Efficacy for Managing Chronic Diseases (SE-CD)
Lee 2021<br>RCT<br>Taiwan	Primary Care; Research Grant and funding from Ministry of Science and Technology	398 patients; 60% female; mean age 73.2; intervention 12 months; follow-up at 3, 6, 9, and 12 months	≥65 years, at least 3 chronic medical conditions	Intervention: Integrated Multidomain Intervention;<br>To determine whether incorporating a multidomain intervention into primary health care could improve standard value-based health outcomes and quality of life;<br>The integrated multidomain intervention entailed 16 2-h sessions per year, comprising communal physical exercise, cognitive training, nutrition and disease education, plus individualised treatment by specialists in integrated geriatric care;<br>Providers: Physical therapist/ fitness coach, occupational therapist/ psychologist, physician;<br>Group-based, in-person; 6 sites in Taiwan;<br>Comparison: Usual Care	HRQoL (SF-36)

Stewart 2021<br>RCT<br>Canada	Primary Care; Research Grant	163 patients; 65.6% female; mean age 62; mean 6 conditions; intervention 4 months; follow-up at 4 months	18 - 80 years, ≥ 3 chronic conditions	Intervention: Telemedicine IMPACT Plus (TIP) programme;<br>Aim: To assess the effectiveness of a provider-created, patient-centred, multi-provider case conference with follow-up, and understand under what circumstances it worked, and did not work;<br>A patient-centred, multi-provider case conference, covering theory, gaps, pre-trial evaluations, and adaptation over time, and contained two key theoretical underpinnings — namely a patient-centred process in the patient–provider interaction and an integrated version of the Chronic Care Model;<br>Providers: Family physician, internist, psychiatrist, social worker, physiotherapist, occupational therapist, pharmacist, dietitian, home care case manager, nurse;<br>Telemedicine; face-to-face (1:1); Nine team-based family practices (primary care sites);<br>Comparison: Usual Care	Self-efficacy for Managing Chronic Disease scale (SEM), Health Education Impact Questionnaire (heiQ)
Mazya 2019<br>RCT<br>Sweden	Community Care; funding from Swedish Government	382 patients; 50.5% male; mean age 82.5; intervention 24 months; follow-up at 24 months	≥ 75 years, had 3 or more current medical diagnoses according to the International Classification of Diseases, 10th Revision (ICD-10)	Intervention: Comprehensive Geriatric Assessment (CGA)-based care and tailored interventions;<br>Aim: To evaluate the effect of outpatient Comprehensive Geriatric Assessment (CGA) on frailty in community-dwelling older people with multimorbidity and high health care utilisation;<br>The Comprehensive Geriatric Assessment and follow up was provided through an outpatient facility that tailors care from a holistic	Number of hospitalisations (published earlier in other papers)

				perspective and, based on each patient's individual needs;<br>Providers: Nurse, social worker, pharmacist, physician, physiotherapist, occupational therapist, dietician;<br>Telemedicine, in-person; Patients' homes or Ambulatory geriatric unit (AGU);<br>Comparison: Usual Care	
Mann 2021*<br>CRT<br>Australia	Primary Care; Funding from Queensland Health and the North Queensland Primary Health Network	80 patients; 55% female; mean age 80.71; intervention 9 months; follow-up at 9 months	Community- dwelling older persons aged 70 or 50 and older if Aboriginal and/or Torres Strait Islander people, having multiple morbidities	Intervention: Community- based integrated care model (OPEN ARCH);<br>Aim: To describes the impact of the OPEN ARCH program on the rate of ED presentations and hospital admissions among OPEN ARCH study participants;<br>A comprehensive, multidimensional geriatric assessment with care coordination performed in a community setting;<br>Providers: GP, geriatric specialist and enablement officer (allied health or nursing);<br>Face-to-face; 14 General Practices<br>Comparison: Usual Care	ED presentations and hospital admissions
Kinchin 2022*<br>Economic evaluation alongside CRT<br>Australia	Primary Care; Funding from Queensland Health and the North Queensland Primary Health Network	80 patients; 55% female; mean age 80.71; intervention 9 months; follow-up at 9 months	Community- dwelling older persons aged 70 or 50 and older if Aboriginal and/or Torres Strait Islander people, having multiple morbidities	Intervention: Community- based integrated care model (OPEN ARCH);<br>Aim: To assess the cost of implementation, delivery and cost-effectiveness (CE) of a flagship community- based integrated care model (OPEN ARCH) against the usual primary care;<br>A comprehensive, multidimensional geriatric assessment with care coordination performed in a community setting;	Health and Social Service Use including emergency department (ED) presentations, hospital admissions, in-patient bed days, allied health and support services utilisation

				Providers: GP, geriatric specialist and enablement officer (allied health or nursing);<br>Face-to-face; 14 General Practices<br>Comparison: Usual Care	
Hong 2021<br>NRSI,<br>USA	Community Care;<br>Research Grant from Abbott Laboratories	1800 patients; 62.5% female; mean age 61.6; intervention 90 days; follow-up at 90 days	≥ 45 years, had 2 or more chronic conditions	Intervention: Quality improvement programme (QIP) Intervention;<br>Aim: To assess outcomes of a nutrition focused quality improvement program (QIP) on healthcare resource use and costs in poorly nourished outpatients;<br>QIP is a nutrition focused, mainly physician-implemented programme on medication needs and on use of healthcare resources;<br>Providers: 5 family or internal medicine physicians, 2 physician assistants, and 2 registered dietitians;<br>In-person, telemedicine; 3 outpatient clinics (Internal Medicine or Family Medicine) of an academic healthcare system in the Los Angeles, California metropolitan area, US;<br>Comparison: historical control; concurrent control	Healthcare resource utilisation, which included the proportion of outpatients presenting for care, and the average number of visits at any of the 3 settings of care
Mateo-Abad 2020a<br>NRSI<br>Six European Regions	Community Care;<br>Institutional funding and grant from the European Commission	856 patients; 51% female; mean age 77.6; intervention 8 - 12 months; follow-up at 8 - 12 months	≥ 65 years, with 2 or more conditions – one of them necessarily being diabetes, congestive heart failure or congestive obstructive pulmonary disease	Intervention: CareWell Programme (An innovative integrated care programme);<br>Aim: To evaluate the impact in terms of use of health services, clinical outcomes, functional status, and patient´s satisfaction of an integrated care program, the CareWell program, for complex patients with multimorbidity;<br>The CareWell integrated care model is based on two main elements: 1) care coordination and	Use of health services - number of contacts with health care providers (general practitioners (GPs), nurses, specialists, and others), number of contacts with social services, number of contacts with the hospital and

				communication between health providers and 2) patient empowerment and home-based care; all supported by ICT-based platforms;<br>Providers: GPs, nurses, specialists (cardiologists, pulmonologists, other specialists), social workers;<br>In-person (Face-to-face), telemedicine; One of the six CareWell sites;<br>Comparison: Usual Care	duration of hospitalizations, and visits to emergency rooms (ER)
Boult 2011<br>CRT<br>USA	Primary Care; Grants from the Agency for Healthcare Research and Quality, the National Institute on Aging, The John A. Hartford Foundation, and contributions from Kaiser-Permanente of the Mid-Atlantic States, Johns Hopkins HealthCare, and the Roger C. Lipitz Center for Integrated Health Care	904 patients; 54.9% female; mean age 77.5; mean 4.3 conditions; intervention 18 months; follow-up at 6 - 18 months	> 65, multimorbid and high service use	Intervention: Guided Care (GC);<br>Aim: To measure the effect of guided care teams on multimorbid older patients' use of health services;<br>Enhanced multidisciplinary team providing self-management support. Home assessments and coordination of care by GC nurses with monthly monitoring over 18 months.<br>Patient care plans and educational materials;<br>Providers: 14 GC nurses, 49 primary care physicians and managing 50-60 patients, training of nurse managers;<br>In-person, telemedicine; Eight primary care systems;<br>Comparison: Usual Care	Health service use
Contant 2019 (Fortin 2016) (secondary analysis of	Primary Care; Fonds Pfizer-Fonds de la Recherche du	281 patients; 50% male; mean age 53.4; mean 5.4 conditions; intervention 3	18 to 75 years of age with at least 3 of the following chronic conditions diabetes,	Intervention: PR1MaC;<br>Aim: To analyse the effect of a multidisciplinary self-management intervention amongst patients with	Self-management (Health Education Impact

multimorbidity subgroup)<br>RCT<br>Canada	Québec en santé (FRQS)-Ministère de la Sante et des Services Sociaux Maladies Chroniques	months; follow-up immediately post-intervention	cardiovascular disease, COPD, asthma, tobacco smoking, obesity and hyperlipidemia	multimorbidity;<br>Initial nurse evaluation with design of individualised intervention plan in collaboration with the patient, based on their objectives; and adaptable over time. Printed information and other educational material for patients. At least 3 individual encounters with trained chronic disease prevention and management (CDPM) professionals over 3 months;<br>Providers: Doctors working together in group practices. Could include encounters with 1 or more CDPM professionals in the following disciplines: nursing, physical activity, nutrition, respiratory therapy and smoking cessation therapy;<br>In-person; Four primary care clinics;<br>Comparison: Usual Care	Questionnaire (heiQ))
Gonzalez Ortega 2017<br>RCT<br>Spain	Primary Care; Grant provided by provided by the Consorci d'Atenció Primària de Salut de l'Eixample	161 patients; 52.8% male; mean age 80.5; mean 3.9 conditions; intervention 6 months; Immediate follow up at intervention completion	Adults with significant chronic disease in 3 or more organ systems	Intervention: Telephone coaching intervention;<br>Aim: To evaluate the impact that adding a telephone coaching intervention by a family physician to usual care has on reducing resource consumption and improving health status, caregiver burden and quality of life amongst complex chronic patients compared with usual care;<br>Telephone coaching and support for self-management by an intervention primary care physician (PCP). Patients had initial face-to-face meeting in their home or in the clinic and were then phoned twice a month over 6 months. Calls addressed symptoms, medications, social contexts and support for self-	Emergency admissions

				management. The PCP also reviewed the patients' record and added notes regarding the calls;<br>Providers: One independent intervention PCP;<br>Face-to-face for first visit and telemedicine for follow-up; Three Primary Care teams;<br>Comparison: Usual care from own PCP	
Hochhalter 2010<br>RCT<br>USA	Primary Care; Grant from the Scott & White Healthcare Research Foundation	79 patients; 65.4% female; mean age 74; mean 3.6 conditions; intervention 3 months; follow-up 3 months after intervention	Adults aged > 65, with ≥ 2 of 7 chronic conditions	Intervention: Patient engagement intervention;<br>Aim: To test the efficacy of a patient engagement intervention for older adults with multiple chronic illnesses;<br>Led by 'coaches' with focus on making most of healthcare, supporting self-management. Checklists and protocols for coaches to follow during the workshop and calls. Two-hour workshop and two telephone calls a week before and a week after a medical appointment.<br>Intervention was designed to prepare patients for appointments, to communicate effectively during appointments and follow through on care plans;<br>Providers: Coaches (professional qualifications and number coaches not reported);<br>Telemedicine; Large Internal Medicine clinic;<br>Comparison: 1. Attention control: 2-h workshop on safety issues and calls before and after a naturally occurring medical encounter. 2. Usual care	Self-management (patient activation measure)
Mercer 2016<br>CRT<br>Scotland	Primary Care; Research Grant from	152 patients; 61% female; mean age 52; mean 4.9 conditions;	≥ 2 long term conditions	Intervention: CarePlus;<br>Aim: To evaluate a whole-system primary care-based complex	Health-related quality of life (EQ-

	Chief Scientist Office of Scottish Government Health Directorates	intervention 12 months; follow-up at 6 and 12 months		intervention, called CARE Plus, to improve quality of life in multimorbid patients living in areas of very high deprivation;<br>Primary care-based whole-system intervention. Structured extended GP consultations and relationship continuity. Practitioner support and training. Patient self-management support with patient support materials;<br>Providers: GP;<br>In-person or telemedicine; Eight general practices in the most deprived parts of Glasgow;<br>Comparison: Usual GP Care	5D-5L) and well-being (W-BQ12)
Salisbury 2018<br>CRT<br>UK	Primary Care; National Institute for Health Research	1546 patients; 51% female; mean age 71; median 3 conditions; intervention 15 months; follow-up at 9 and 15 months	18 years or older, with ≥ 3 chronic conditions, based on 17 chronic conditions in Quality and Outcomes Framework	Intervention: 3D intervention;<br>Aim: To implement and assess the effectiveness of a new approach to managing patients with multimorbidity in primary care;<br>3D intervention based on patient centred care with focus on continuity, coordination, and efficiency of care with 6-monthly comprehensive multidisciplinary review (nurse, pharmacist and physician/ GP) with extended appointments if requested. IT support to facilitate identification of patients, recall and 3D templates. Printed care plans to support shared decision making. Practice training: 2 half-days Practice supports: nominated practice 3D champion, automated monthly feedback compared to peers and financial incentives for completed reviews (GBP 30 per review);<br>Providers: GP, practice nurse and pharmacists (who may or may not	Health-related quality of life (EQ-5D-5L)

				have worked with the practice previously);<br>In-person; 33 general practices;<br>Comparison: Usual GP Care	
Schafer 2018<br>CRT<br>Germany	Primary Care; Grant from the German Federal Ministry of Education and Research	650 patients; 50.5% female; mean age 73.3; mean 8.7 conditions; intervention 12 months; follow-up at 12 months with final data collection at intervention completion	≥ 3 conditions	Intervention: Multicare AGENDA;<br>Aim: To determine if patient-centred communication leads to a reduction in the number of medications taken without reducing health-related quality of life;<br>Patient-centred communication GP Training: 3 sessions lasting 4 h on narrative based patient-doctor dialogues. Three 30 min 'talks' between GP and patients over 12 months: 1. Focus on patient priorities (including non-medical) 2. Medication review 3. Review previous goals and considered goal attainment at end of 12 months;<br>Providers: GP<br>In-person; 55 general practices;<br>Comparison: Usual care with wait-list control	Number medications and Health-related quality of life (EQ-5D)
Sommers 2000<br>RCT<br>USA	Primary Care; Locally funded	543 patients; 70% female; mean age 77.5; Intervention 18 months, follow-up 12 months after intervention	> 65, with at least 2 conditions	Intervention: Senior Care Connections;<br>Aim: To examine the impact of an interdisciplinary, collaborative, practice intervention for community dwelling seniors with chronic illnesses;<br>Enhanced multidisciplinary teams with 2 months immersion in primary care practice for the nurses and social workers before intervention commenced. Initial home assessment by the nurse or social worker to gather data on patient concerns. Team then met and drafted risk reduction care plans and support	Health service use and self-rated health

				for self-management to discuss with patients and family members. Nurse or social worker monitored patients every 6 weeks between primary care physicians (PCP visits) either in home, in clinic or by phone. Monthly team meetings to discuss patient progress with training and ongoing support for nurses and social workers;<br>Providers: 18 PCPs working in 9 teams with a full-time nurse with geriatrics training and half-time social worker per team;<br>In-person and telemedicine; home or clinic;<br>Comparison: Usual Care	
Kiely 2024<br>RCT<br>Ireland	Primary Care; Health Research Board Ireland and Department of Health Sláintecare Integration Fund	251 patients; 63% female; mean age not reported, 59% < 65 years; intervention 1 month; follow-up at 1 month	⩾ 18 years, 2 or more chronic conditions, 5 or more medications	Intervention: LinkMM intervention;<br>Aim: To explore the feasibility, potential impact on health outcomes and cost effectiveness of practice-based link workers for people with multimorbidity living in deprived urban communities;<br>Primary care practice-based social prescribing link workers on health outcomes and costs for people with multimorbidity;<br>Providers: Link workers at 13 general practices;<br>In-person, telephone; 13 general practices;<br>Comparison: Wait-list usual care	Health related quality of life (EQ-5D-5L), mental health (Hospital Anxiety and Depression Scale)

* Gillespie 2025 is the secondary economic paper based on the main study of McCarthy 2022; Gillespie 2022 is the secondary economic paper based on the main study of O'Toole 2021; Kinchin 2022 is the secondary economic paper based on the main study of Mann 2021

Tab. 2 Results of primary and secondary outcomes reported in each of the included studies

Study ID, Study Design, Study Location (Country)	Primary Outcome: Results	Secondary Outcome: Results
**Medicines management**		
Yang 2022<br>RCT<br>Hong Kong, China	Medication adherence:<br>Statistically significant improvement was found in medication adherence in intervention group as compared to the control group immediately post-intervention (β = 1.63, p = 0.034). Improved medication adherence was observed in the intervention group at 3 months follow-up compared with baseline, although the differences between the groups were not significant.	The intervention group demonstrated significantly greater improvements in medication knowledge (β = 2.26, p < 0.001), beliefs about necessity of medication (β = 2.68, p = 0.002) and concerns about medication (β = −2.44, p = 0.002), medication self-efficacy (β = 1.87, p = 0.015) and medication burden (β = −3.96, p = 0.004) as compared with the control group at 3 months follow-up.<br><br>No statistically significant effects were found on quality of life and utilisation of health care services at both time points.
Jager 2017<br>CRT<br>Germany	Summary score of 10 prescribing indicators:<br>The increase in the degree of implementation was 4.2 percentage points (95% CI – 0.3 to 8.6) higher in the intervention group compared to the control group (p = 0.1).	Harms were not expected or reported.<br>No significant difference in Patient Activation Measure (P AM-13D); Medication Adherence Report Scale (MARS); Beliefs About Medicines Questionnaire (BMQ-D) and % Potentially Inappropriate Medicines (PIMs).
Koberlein Neu 2016<br>CRT<br>Germany	Quality of medication therapy [mean Medication Appropriateness Index (MAI) score]:<br>Mean MAI score: Intervention phase 1 vs Control Phase, aMD<br>4.51, 95% CI 6.66 to 2.36.	Mean reduction in drug-related problems of – 0.45, 95% CI – 0.81 to – 0.09, P = 0.014.<br>No significant difference in Number of drug-related problems (DRPs); Potentially inadequate medication (PIM); Number of prescribed medicines per patient; HRQoL (SF12); Function (Barthel Index); Instrumental Activities of Daily Living (iADL); Gait stability/risk of falling (Tinetti score).<br>Level of social support results not reported.
Krska 2001<br>RCT<br>UK	Pharmaceutical care issues:<br>Pharmaceutical care issues (%) resolved after intervention: 82.7% v 41.2%, p < 0.001.	No significant differences in medication costs, HRQoL (SF36 scores), and health & social services use.
Muth 2018<br>CRT<br>Germany	MAI score at 6 months:<br>No significant effect on mean MAI sum scores with aMD of 0.7 (95% CI – 0.2 to 1.6).	Functional status (Vulnerable Elderly Survey-13) MD 0.4, 95% CI 0.0 to 0.8, p = 0.047<br>No significant difference in all other secondary outcomes including MAI at 9 months; HRQoL EQ-5D (aMD 2.3; 95% CI – 1.6 to 6.2, p = 0.247); All-cause hospitalisation; Severity of chronic pain (von Korf Index); Satisfaction with shared decision-making (Man-Son-Hing Scale); Patient's future expectation, expected/ desired lifetime duration; Years of

		Desired Life (YDL); Medication adherence: Observed adherence: drug score, dose score, regimen score; Self-reported adherence (Morisky); Patient Beliefs about Medicines Questionnaire (BMQ); Medicines prescribed; Medication Regimen Complexity Index and number of prescriptions/single doses
McCarthy 2022*<br>CRT<br>Ireland	Number of medicines:<br>Intervention vs control group at follow-up, there was a small but significant effect (IRR 0.95, 95% CI: 0.899 to 0.999, p = 0.045)<br><br>Adjusted odds of having a PIP:<br>Intervention group vs control group at follow-up (OR 0.39, 95% CI: 0.140 to 1.064, p = 0.066)	Significantly more medicines stopped and a significant reduction in the odds of being prescribed ≥15 medicines in the intervention compared to the control group at follow-up. There was no evidence of an effect demonstrated on any of the PIP-related or patient-reported outcome measures or on healthcare utilisation. There was a reduction in the number of GP visits and an increase in the number of telephone consultations in both groups at follow-up
Gillespie 2025*<br>Economic evaluation alongside CRT<br>Ireland	SPPiRE intervention was the dominant strategy over usual care.<br>The probability that the SPPiRE intervention is cost effective was 0.993 and 0.988 at threshold values of €20,000 and €45,000 per QALY respectively	Secondary outcomes were not available in this study.
**Support for self-management**		
Gillespie 2022*<br>Economic evaluation alongside RCT<br>Ireland	Health-related quality of life (EQ-5D-3L):<br>Mean EQ-5D-3L score after 6 months follow-up was 0.51 (SD: 0.34) in the intervention group while it was 0.35 (SD: 0.38) in the usual care group.<br>Cost-effectiveness Results:<br>Cost: The intervention was associated with a reduction in mean costs of €2,548 (P-value: 0.114; 95% CIs: −€5606 to €509) per patient.<br>QALY: The intervention was associated with an increase in mean QALYs of 0.031 (P-value: 0.063; 95% CI: −0.002 to 0.063) per patient.<br>At alternative threshold values of €5,000, €10,000, €20,000, €30,000, and €45,000, the probability of the intervention being cost-effective was estimated to be 0.945, 0.948, 0.951, 0.955, and 0.958, respectively.	Secondary outcomes were not available in this study.
Khunti 2021<br>RCT<br>UK	Physical activity (PA):<br>Reduction in overall daily PA was greater in the intervention group compared with the control group at 12 months (–0.80 milligravity; 95% CI = –1.57 to –0.03; P = 0.04).	PA-related secondary outcomes:<br>A relative reduction in time spent undertaking moderate–vigorous PA (–3.86 min per day; 95% CI = –6.70 to –1.03; P = 0.008) and time spent at an intensity equivalent to a slow walk

		(–4.66 min per day; 95% CI = –8.82 to –0.51; P = 0.028) in the intervention group<br>at 12 months.<br>No other statistically significant findings were observed.<br><br>Anthropometric and clinical measures:<br>No statistically significant differences between groups were observed in anthropometric or clinical measures.<br><br>EuroQol five-dimensional, five-level version (EQ-5D-5L):<br>A statistically significant reduction was found in the intervention group in relative to control at 12 months in self-rated health (visual<br>analogue scale [VAS]) from the EQ-5D- 5L (–3.85; 95% CI = –7.60 to –0.09; P = 0.045) in the intervention group.<br><br>No statistical differences were observed in other measures.
Lear 2021<br>RCT<br>Canada	Number of all-cause hospitalisations:<br>25 fewer all-cause hospitalizations occurred in the intefvention group vs the usual care group (56 hospitalizations vs 81 hospitalizations; 30.9% reduction).<br>No statistical difference in hospitalisation between two groups (relative risk [RR], 0.68; 95% CI, 0.43-1.10; P = 0.12).	In-hospital days:<br>229 fewer in-hospital days occurred in the intervention group vs the usual care group (282 days vs 511 days).<br>No statistical difference in the number of in-hospital days between two groups (RR, 0.52; 95% CI, 0.24-1.10; P = 0.09).<br><br>HRQoL (measured by the Medical Outcomes Study 36-item Short Form survey [SF-36]):<br>No differences were observed between the two groups.<br><br>Self-management (measured by Health Education Impact Questionnaire [heiQ]):<br>Significant changed in favour of the intervention group in 4 of the 8 domains: skill and technique acquisition, self-monitoring and insight, social integration and support, and emotional well-being.
O'Toole 2021*<br>RCT<br>Ireland	HRQoL (EQ-5D-3L), HRQoL (EQ-VAS); Frequency of activity participation (FAI):<br>No differences in primary outcomes at 6-month follow-up.<br>EQ-VAS aMD = 6.07 (95% CI – 1.77 to 13.91)<br>EQ-5D-3L index score aMD = 0.10 (95% CI – 0.01 to 0.22)<br>FAI aMD = 1.20 (95% CI – 0.89 to 3.29)	Statistically significant difference was seen in perceptions of occupational satisfaction (COPM-S) in the intervention compared with the control group (aMD = 1.24; 95% CI 0.43 to 2.06).

		Significant difference was seen in favour of the intervention group in hospital outpatient appointments (adjusted MD = –1.69; 95% CI –2.66 to –0.72).<br><br>No evidence of significant differences were seen in other secondary outcomes examined at 6 months.
Shang-Lin 2020<br>RCT<br>Taiwan	Physical activity amount; HRQoL (SF-36):<br>Experimental group had higher amounts of physical activity (β = 1333, P = 0.004) and moderate-intensity physical activity (β = 330, P = 0.04) than the control group at 12 weeks after the intervention.<br><br>Experimental group had increased exercise capacity (VO_{2peak}, β = 4.43, P = 0.04)<br><br>Improvement in health-related quality of life (physical function, β = 7.55, P = 0.03; and physical component summary, β = 4.42, P = 0.03) was seen in the experimental group as compared with those in the control group.	Secondary outcomes were not specified in this study.
Sturm 2022<br>CRT<br>Germany	Self-Efficacy for Managing Chronic Disease 6-Item Scale (SES6G):<br>No significant difference between intervention and control groups (MD = 0.30, 95% CI – 0.21 to 0.81, P = 0.25).	Marginal effect on mental well-being measured by the SF12 questionnaire (MD = 3.34, 95% CI 0.99 to 5.69, P = 0.006).<br><br>No significant difference between intervention group and control group in SF12 (physical well-being).
Eakin 2007<br>RCT<br>USA	Dietary behaviour and physical activity:<br>Adjusted means (SE) at 6 months post-intervention:<br>Dietary behaviour (lower score better) intervention 2.24 (±0.05) vs usual care 2.41 (±0.05), p < 0.05; change minutes walking/week intervention 16 (±20) vs usual care -11 (±23), p > 0.5	Multilevel support for healthy lifestyle (higher score better) 2.98 (±0.06) v 2.69 (±0.05), p < 0.05
Garvey 2015<br>RCT<br>Ireland	Activity participation (Frenchay Activities Index):<br>aMD at immediate follow up 4.22, 95% CI 1.59 to 6.85	Significant improvements in perceptions of activity performance and satisfaction, self-efficacy, independence in daily activities, HRQoL (EQ-5D VAS scores only), and HeiQ score (Positive and active engagement in life only).<br>The intervention group demonstrated significantly higher levels of goal achievement, following the intervention.<br>No significant differences in anxiety, depression, other domains of HeiQ scores, and healthcare utilisation.
Reed 2018<br>RCT	Self-rated Health:	No significant differences in Fatigue; Pain; Health distress; Energy; Depression; Illness intrusiveness; Exercise;

Australia	Intervention participants were more likely than control participants to report improved self-rated health at 6 months: Odds Ratio (2.50, 95% CI, 1.13 to 5.50, p = 0.023).	Medication adherence; Self-Efficacy; Health Education Impact (HEiQ); Healthcare utilisation (GP visits, Emergency Department (ED) visits and admissions).
Tew 2024<br>RCT<br>England and Wales	EQ-5D-5L utility index score:<br>There was an adjusted mean difference of 0.020 favouring intervention (95% CI −0.006 to 0.045, p = 0.14) but no statistically significant difference.	No statistically significant differences were observed in secondary outcomes, except for the pain items of the Patient-Reported Outcomes Measurement Information System-29.
**Care coordination plus support for self-management**		
Chan 2023<br>RCT<br>USA	Change in hospitalisation rate:<br>Six-month hospitalisations decreased in both the A-ICU and EUC<br>groups, with no difference between them (mean [SE], −0.6 [0.5] vs −0.9 [0.5]; difference, 0.3 [95% CI, −1.0 to 1.5]).	Emergency department use did not differ between groups (mean [SE], −2.0 [1.0] vs 0.9 [1.0] visits per person; difference, −1.1 [95% CI, −3.7 to 1.6]). Primary care physician visits increased in the A-ICU group (mean [SE], 4.2 [1.6] vs −2.0 [1.6] per person; difference, 6.1 [95% CI, 1.8 to 10.4]). Patients in the A-ICU group reported improved in self-rated health (mean [SE], 0.7 [0.3] vs −0.2 [0.3]; difference, 1.0 [95% CI, 0.1 to 1.8]) compared with patients in the EUC group.
Fisher 2020<br>RCT<br>Canada	HRQoL (SF-12), Physical Component Summary (PCS):<br>There was no evidence of a significant difference between study groups in the primary outcome, PCS, with a mean difference of – 4.94 (95% CI: – 12.53 to 2.66, p = 0.20).	HRQoL (SF-12), Mental Component Summary (MCS):<br>No significant differences between groups were seen for MCS and some other secondary outcomes including the Centre for Epidemiologic Studies Depression Scale (CESD-10), the Generalized Anxiety Disorder Scale (GAD-7), and Self-efficacy measured using the Self-Efficacy for Managing Chronic Disease Scale.<br><br>The proportion of hospitalisations during the period 6 months prior to baseline (0.56) was significantly higher compared to the proportion of hospitalisations during the 6-month intervention period (0.06) for intervention group (p = 0.01). No significant difference was seen for the control group (p = 0.08).<br><br>No significant difference in ED visits for the intervention group (p = 0.27), whereas a significant difference was found for the control group (p = 0.02) indicating that the proportion of ED visits during the period 6 months prior to baseline (0.81) was significantly higher compared to the proportion of ED visits during the 6-month intervention period (0.25).<br><br>The only service cost showing a statistically-significant difference between the two groups was the Home Care & Outpatient service costs.

Fortin 2021<br>RCT<br>Canada	Health Education Impact Questionnaire (heiQ) and Self-Efficacy for Managing Chronic Diseases (SE-CD):<br>Among the 8 domains of the heiQ, only 1 showed a statistically significant difference favouring the intervention group, which is self-monitoring and insight with mean difference 0.23; p < 0.01. Overall, the intervention showed a neutral effect for the primary outcomes.	Health status measured by the validated Veterans RAND 12 Item Health Survey (VR-12):<br>Health status and quality of life did not appear to be affected by the intervention. There was significant improvement in 2 health behaviours (healthy eating with odds ratios [OR] 4.36; p = 0.006, and physical activity with OR 3.43; p = 0.023).
Lee 2021<br>RCT<br>Taiwan	HRQoL (SF-36):<br>The integrated multidomain intervention group had significantly higher mean SF-36 physical component scores across all timepoints as compared to the usual care group (overall difference 0.8, 95% CI 0.2–1.5; p=0.010), but differences at 3, 6, 9, and 12 months did not reach statistical significance. The SF-36 mental component scores did not differ significantly overall, but were significantly higher in the integrated multidomain intervention group at the 12-month follow-up (55.3 [SD 7.6] vs 57.2 [7.0]; p=0.019).	ICHOM Standard Set for Older Person:<br>Compared with usual care, the integrated multidomain intervention group had significantly higher mean ICHOM value-based metric scores (overall difference 0.2, 95% CI 0.1–0.3; p=0.0031) and significantly higher scores at 12 months (7.2 [SD 1.4] vs 7.6 [1.4]; p=0.049).
Stewart 2021<br>RCT<br>Canada	Self-efficacy for Managing Chronic Disease scale (SEM), Health Education Impact Questionnaire (heiQ):<br>No statistically significant differences were shown on both the primary outcomes.	VR12 Health Status, EQ-5D quality of life, Kessler Psychological Distress Scale, and Health Behaviour Survey:<br>No statistically significant differences were shown on all the secondary outcomes.
Mazya 2019<br>RCT<br>Sweden	Number of hospitalisations (published earlier in other papers).	Frailty:<br>Intervention group contained a significantly greater proportion of pre-frail individuals than the control group (p = 0.004). The proportion of frail and deceased combined was also significantly lower in the intervention group (p = 0.002). Mortality rates were high; 35 (18%) participants in the intervention group and 42 (26%) participants in the control group died, with no significant difference between groups (p = 0.051).
Mann 2021*<br>CRT<br>Australia	No effect of the intervention on ED presentations (Model 1–IRR = 0.91, 95%CI 0.56–1.47, p = 0.697). This result remained stable after adjusting for time period (Model 2– IRR = 1.35, 95%CI 0.57–3.17, p = 0.498) and demographics (Model 3–IRR = 1.17, 95%CI 0.52–2.66, p = 0.703).<br><br>A similar trend was observed for hospital admissions.	Secondary outcomes were not specified in this study.
Kinchin 2022*	Health and Social Service Use including emergency department (ED) presentations, hospital admissions, in-	Health- related quality of life assessed using EQ-5D-3L and AQoL- 8D:

Economic evaluation alongside CRT Australia	patient bed days, allied health, and support services utilisation:<br>No statistically significant differences were observed in the resource use between intervention and usual care phases. Inpatient discharges and a corresponding average length of stay were lower in the intervention phases than in the usual care phases, although not statistically significant.<br><br>Functional Independence Measure (FIM):<br>Although not statistically significant, FIM showed improvement in the intervention group.	No statistically significant differences were observed in the effect measures between intervention and usual care phases.<br><br>Cost-effectiveness analysis:<br>Outcome 1 – Functional Independence Measure (FIM)<br>ICER was US$535 per improvement of one point on the FIM Scale from the health system perspective whereas it was US$548 from the health system and personal perspective.<br><br>Outcome 2 – Inpatient stay<br>The estimated ICER for inpatient stay was US$9597 from the health system perspective while the ICER for inpatient stay from the health system and personal perspective was US$9528.<br><br>Outcome 3 – Average length of stay (ALOS)<br>The estimated ICER for ALOS was US$1922 per day reduction from the health system perspective and US$1876 per day reduction from the health system and personal perspective.
Hong 2021<br>NRSI,<br>USA	Healthcare resource utilisation, which included the proportion of outpatients presenting for care, and the average number of visits at any of the 3 settings of care:<br>The proportion of QIP outpatients presenting for healthcare services<br>was significantly reduced compared to both historical and concurrent controls—relative risk reduction (RRR) versus historical (11.6%, $P < .001$) and versus concurrent (8.9%, $P = .003$).<br><br>Patients in the QIP group had significantly fewer visits, as compared to the historical (12.9%, $P = .022$) control group but not the concurrent<br>(0%, $P = .977$) control group.	Medication utilisation over 90-days:<br>Medication usage remained constant from baseline to 90 days for the QIP group (5.81 vs 5.80, −0.2%, $P = .878$), while medication usage in the control groups increased significantly over the 90-day period (historical group 8.50 vs 8.83, +3.9%, $P = .001$; concurrent<br>group 7.81 vs 8.35, +6.9%, $P = .001$).
Mateo-Abad 2020a<br>NRSI<br>Six European Regions	Use of health services - number of contacts with health care providers (general practitioners (GPs), nurses, specialists, and others), number of contacts with social services, number of contacts with the hospital and duration of hospitalisations, and visits to emergency rooms (ER):<br>The number of visits to ER services was significantly lower in the intervention group ($p = 0.001$) but the number of visits	Secondary outcomes were not available in this study.

	to GPs and primary care nurses increased. The mean length of hospital stay among those who had been hospitalized was shorter in the intervention group (p = 0.033). No differences were observed for the use of social services.<br><br>There was no significant effect of the CareWell program on clinical outcomes.	
Boult 2011<br>CRT<br>USA	Health service use:<br>Adjusted ratio of service use: hospital admissions 1.01 (95% CI 0.83 to 1.23); 30-Day readmissions 0.79 (0.53 to 1.16); hospital days 1.00 (0.77 to 1.30); skilled nursing facility admissions 0.92 (0.6 to 1.4); skilled nursing facilities days 0.84 (0.48 to 1.47); emergency department visits 1.04 (0.81 to 1.34); primary care visits 1.02 (0.91 to 1.14); speciality care visits 1.07 (0.93 to 1.23); home healthcare episodes 0.70 (0.53–0.93).	PACIC (Patient Assessment of Chronic Illness Care) score:<br>PACIC score at 18 months adjusted mean difference (aMD) 0.2 95% CI 0.07 to 0.33, p = 0.002.<br>Satisfaction: no difference between groups.<br>Provider satisfaction with care mixed effects.
Contant 2019 (Fortin 2016) (secondary analysis of multimorbidity subgroup)<br>RCT<br>Canada	Self-management (Health Education Impact Questionnaire (heiQ) 8 domains):<br>The intervention group showed improvement in 4 of the 8 heiQ domains in multivariate analysis: These four domains were: health directed behaviour: OR 1.98, 95% CI 1.07 to 3.66, p = 0.029; constructive attitudes and approaches: OR 3.92, 95% CI 1.73 to 8.89, p = 0.001; skill and technique acquisition OR 2.48, 95% CI 1.32 to 4.65, p = 0.005; health service navigation OR 2.73, 95% CI 1.2 to 6.22, p = 0.02. There were no significant improvements in positive and active engagement in life, emotional well-being, self-monitoring and insight, and social integration and support.	Secondary outcomes were not reported in this secondary data analysis study of Fortin 2016.
Gonzalez Ortega 2017<br>RCT<br>Spain	Emergency admissions:<br>After 6 months, urgent visits per patient decreased in intervention 1.27 baseline versus 0.89 follow-up, p = 0.091 and control 1.06 baseline versus 0.86 follow-up, p = 0.422, mean difference 0.18 [95% CI – 0.48 to 0.84].	HRQoL SF12:<br>There was a significant effect on physical component score (aMD – 4.71, 95% CI – 9.03 to – 0.41, p = 0.033) but no effect on the mental component score (aMD 2.60, 95% CI – 3.9 to 9.11, p = 0.424).<br>No significant effect on clinic visits; Charlson score; Function (Barthel); HRQoL; Cognitive status (Pfeiffer test); Pressure Ulcer risk (Norton scale); Social risk (Gijon Test); Caregiver Burden (Zarit test); chronic treatment (number of repeat medicines) or resource use (direct costs).
Hochhalter 2010<br>RCT	Self-management – patient activation measure (PAM):	Self-efficacy:

USA	PAM Intervention 66.8 (18.5) vs Control 66.2 (13), no significant difference, all groups had significant improvement from baseline	Significant improvement in self-efficacy compared to usual care (but attention control group also had a significant improvement).<br><br>No difference in total unhealthy days and self-rated health.
Mercer 2016<br>CRT<br>Scotland	Health-related quality of life (EQ-5D-5L) and well-being (W-BQ12):<br>EQ5D Index scores: 0.06 (95% CI – 0.02 to 0.14, p = 0.15)<br>EQ-5D-5L area under the curve over the 12 months was higher in the CARE Plus group (p = 0.002).<br>CARE Plus significantly improved one domain of well-being (negative well-being), with an effect size of 0.33 (95% confidence interval [CI] 0.11–0.55) at 12 months (p = 0.0036). Positive well-being, energy, and general well-being (the combined score of the three components) were not significantly influenced by the intervention at 12 months.	Anxiety and depression (Hospital Anxiety and Depression Scale, HADS), self-efficacy and self-esteem:<br>No significant difference in anxiety and depression (HADS); self-efficacy, self-esteem, and medications.<br>Cost Effectiveness Analysis: Within-trial cost-utility analysis based on the EQ-5D-5L utility scores, and on health service utilisation: Adjusted mean difference in cost of GBP929 (95% CI 86 to 1788) per patient<br>Gain in QALY 0.076 (95% CI 0.028-0.124)<br>Cost effectiveness ratio (CER) GBP12,224 per QALY
Salisbury 2018<br>CRT<br>UK	Health-related quality of life (EQ-5D-5L):<br>No difference between groups with EQ-5D-5L aMD 0.00, 95% CI – 0.02 to 0.02; p = 0.93.	PACIC score: aMD 0.29 (95% CI 0.16 to 0.41); p < 0.0001.<br>Continuity of care score: adj MD 0.08; 95% CI 0.02 to 0.13; p = 0.0045.<br>Mean Consultation and Relational Empathy (CARE) score (for doctor consultations): aMD 1.2; 95% CI 0.28 to 2.13; p = 0.0109.<br>Mean CARE score (for nurse consultations): aMD 1.11; 95% CI 0.03 to 2.19; p = 0.044.<br>Higher proportion of intervention patients were very satisfied with their care (56%) compared to those receiving usual care (39%) (MD 1.57, 95% CI 1.19 to 2.08, p = 0.0014).<br>No significant differences in Self-rated health; Bayliss measure of illness burden; depression and anxiety (HAD scale); Treatment burden (MTBQ); Medication adherence (Moriskey measure) and number of medications; Number high risk prescriptions; Healthcare utilisation (GP and nurse visits, OPD visits and admissions) and Quality of care (QOF indicators).<br>Cost-effectiveness: 50.8% chance of being cost-effective at a willingness-to-pay threshold of GBP20 000 per QALY (55.8% at £30 000 per QALY). Reported as ‘equivocal cost-effectiveness’.
Schafer 2018<br>CRT<br>Germany	Number medications and Health-related quality of life (EQ-5D):	Increase in prescribing of analgesics in the intervention group (Adjusted RR 2.043, P = 0.019).

	No difference between groups in the change of the number of medications taken: 0.43, 95% CI – 0.07 to 0.93; p = 0.094. No difference in EQ-5D index score: 0.34; 95% CI – 0.05 to 0.74; p = 0.091.	No significant differences in patient satisfaction; patient empowerment; depression; healthcare utilisation or in direct costs reported using Leipzig supply and Cost Instrument.
Sommers 2000<br>RCT<br>USA	Health service use and self-rated health:<br>Odds ratio admissions/patient/year 0.63 (95% CI 0.41 to 0.96); ⩾ 1 60-day readmissions 0.26 (0.08 to 0.84).<br>Not fully reported for seven other outcomes, non-significant for five. Difference in adjusted mean scores, social activities count 0.50 (95% CI 0.02 to 1.00). Symptom scale 0.50 (– 3.20 to 0.16), SF-36 self-rated health 0.10 (– 0.27 to 0.02), not reported for four other outcomes, non-significant.	Social activities count: Int = 0.2 vs Con – 0.3, p = 0.04.<br>No significant differences in patient reported health status; social activities count; HRQoL (SF36); depression scores; nutrition checklists and drug adherence.
Kiely 2024<br>RCT<br>Ireland	Health related quality of life (EQ-5D-5L) and mental health [Hospital Anxiety and Depression Scale (HADS)]:<br>There were no significant differences identified using mixed effects regression analysis in EQ-5D-5L (MD 0.01, 95% CI -0.07 to 0.09) or HADS (MD 0.05, 95% CI -0.63 to 0.73).	No significant differences were found in wellbeing measured by ICEpop CAPability measure for Adults (ICECAP-A), frequency of activity participation measured by Frenchay Activity Index (FAI), and self-management behaviour measured by Patient Activation Measure (PAM).

* Gillespie 2025 is the secondary economic paper based on the main study of McCarthy 2022; Gillespie 2022 is the secondary economic paper based on the main study of O'Toole 2021; Kinchin 2022 is the secondary economic paper based on the main study of Mann 2021

Tab. 3 Summary of secondary outcomes

Study ID	Outcomes	Treatment effects, intervention vs usual care [Mean (SD) or MD, 95% CI unless stated otherwise]
**Medicine management**		
Yang 2022	Medication adherence (MARS-5), immediately post-intervention	β-coefficient, (95% CI), 1.63 (0.12, 3.15); p = 0.03
	Medication self-efficacy (SEAMS), immediately post-intervention	β-coefficient, (95% CI), 3.22 (1.68, 4.77); p = <0.001
	Medication self-efficacy (SEAMS), 3 months follow up	β-coefficient, (95% CI), 1.87 (0.37, 3.38); p = 0.02
Jager 2017	Summary score of 10 prescribing indicators	31.50 (8.00) vs 27.90 (6.50); p = 0.10
	Medication adherence (MARS-5)	22.30 (3.30) vs 23.30 (2.60); p = 0.11
	Number of PIM prescriptions per year	0.80 (1.80) vs 1.00 (1.90); p = 0.37
Koberlein Neu 2016	MAI sum score per patient	–4.51 (–6.66 to –2.36); p < 0.001
	Number of DRP per patient	–0.45 (–0.81 to –0.09); p = 0.01
	Number of PIM prescribed	–0.04 (–0.09 to 0.01)
Krska 2001	Total pharmaceutical care issues resolved, percentage	78.8% vs 39.3%
Muth 2018	MAI	0.70 (–0.20 to –1.60); p = 0.14
	Self-reported adherence	0.00 (–0.20 to 0.10); p = 0.63
	Observe adherence: drug score	OR: 0.90 (0.60 to 1.40); p = 0.74
	Observe adherence: dose score	OR: 1.40 (0.90 to 2.00); p = 0.12
	Observe adherence: regimen score	OR: 1.40 (0.90 to 2.10); p = 0.15
	Number of prescriptions	RR: 1.00 (1.00 to 1.10); p = 0.31
	MRCI	1.00 (−0.60 to 2.50); p = 0.21
	Satisfaction with shared decision-making (Man-Son-Hing Scale)	−0.20 (−1.00 to 0.50); p = 0.52
McCarthy 2022	Number of medicines stopped	IRR: 1.48 (1.171 to 1.871); p = 0.001
	Number of medicines started	IRR: 1.12 (0.826 to 1.513); p = 0.470
	Proportion prescribed ≥15 medicines	OR: 0.37 (0.193 to 0.719); p = 0.003
	Number of PIP	IRR: 0.92 (0.813 to 1.057); p = 0.256
	Proportion with any reduction in PIP	OR: 1.42 (0.892 to 2.255); p = 0.140
	Proportion with at least 1 high-risk PIP	OR: 0.93 (0.528 to 1.642); p = 0.806
**Support for self-management**		
Khunti 2021	Overall daily physical activity	–0.80 (–1.57 to –0.03); p = 0.04
Lear 2021	heiQ (Positive and Active engagement in life)	Medians (25th and 75th percentiles), 5.00 (4.40,5.40) vs 4.80 (4.00,5.00); p = 0.05
	heiQ (Health-directed behaviour)	Medians (25th and 75th percentiles), 4.50 (3.50,5.30) vs 3.80 (2.80,5.00); p = 0.06
	heiQ (Skill and technique acquisition)	Medians (25th and 75th percentiles), 5.00 (4.40,5.20) vs 4.60 (4.00,5.00); p < 0.01
	heiQ (Constructive attitude shift)	Medians (25th and 75th percentiles), 5.00 (4.80,5.80) vs 5.00 (4.60,5.40); p = 0.11
	heiQ (Self-monitoring and insight)	Medians (25th and 75th percentiles),

		5.10 (5.00,5.60) vs 5.00 (4.70,5.40); p = 0.02
	heiQ (Health service navigation)	Medians (25th and 75th percentiles), 5.20 (5.00,6.00) vs 5.00 (5.00,5.80); p = 0.18
	heiQ (Social integration and support)	Medians (25th and 75th percentiles), 5.00 (4.60,5.40) vs 5.00 (4.00,5.40); p = 0.02
	heiQ (Emotional Wellbeing)	Medians (25th and 75th percentiles), 5.00 (4.50,5.50) vs 4.70 (3.70,5.20); p < 0.01
O'Toole 2021	FAI	1.20 (–0.89 to 3.29); p = 0.257
	NEADL	1.84 (–0.89 to 4.58); p = 0.184
	SEMCD	0.52 (0.00 to 1.05); p = 0.052
Sturm 2022	SES6G	0.30 (−0.21; 0.81); p = 0.25
	MARS	0.08 (−0.28; 0.44); p = 0.65
	GSES	0.16 (−0.89; 1.21); p = 0.81
Eakin 2007	Dietary behaviour	Mean (SE), 2.24 (±0.05) vs 2.43 (±0.05); p < 0.05
	Multilevel support for healthy lifestyles	Mean (SE), 2.98 (±0.06) vs 2.69 (±0.05); p < 0.05
	Change minutes of walking/week	Mean (SE), 16 (±20) vs –11 (±23); p > 0.05
Garvey 2015	FAI	4.22 (1.59 – 6.85); p < 0.01
	SEMCD	6.79 (1.51) vs 5.32 (1.92); p = 0.02
	NEADL	47.18 (11.87) vs 40.73 (10.71); p = 0.02
	heiQ (Positive and Active engagement in life)	2.93 (0.63) vs 2.62 (0.56); p = 0.04
	heiQ (Health-directed behaviour)	3.04 (0.69) vs 2.73 (0.81); p = 0.99
	heiQ (Skill and technique acquisition)	3.04 (0.50) vs 2.78 (0.34); p = 0.99
	heiQ (Constructive attitudes and approaches)	3.01 (0.62) vs 2.95 (0.41); p = 0.99
	heiQ (Self-monitoring and insight)	3.25 (0.39) vs 2.97 (0.44); p = 0.99
	heiQ (Health service navigation)	3.15 (0.46) vs 3.05 (0.51); p = 0.99
	heiQ (Social integration and support)	3.01 (0.75) vs 2.84 (0.57); p = 0.99
	heiQ (Emotional Wellbeing)	2.35 (0.70) vs 2.23 (0.70); p = 0.99
Reed 2018	Walk for exercise	8.86 (–7.43; 25.15); p = 0.29
	MARS-5	0.20 (–0.09 ; 0.50); p = 0.18
	heiQ (Positive and Active engagement in life)	–0.03 (–0.12; 0.05); p = 0.48
	heiQ (Health-directed activities)	0.03 (–0.09; 0.15); p = 0.64
	heiQ (Skill and technique acquisition)	–0.03 (–0.11; 0.05); p = 0.46
	heiQ (Constructive attitudes and approaches)	0.01 (–0.08; 0.10); p = 0.82
	heiQ (Self-monitoring and insight)	0.01 (–0.09; 0.07); p = 0.82
	heiQ (Health service navigation)	0.06 (–0.03; 0.15); p = 0.18
	heiQ (Social integration and support)	–0.03 (–0.12; 0.07); p = 0.58
	heiQ (Emotional distress)	0.02 (–0.07; 0.12); p = 0.61
**Care coordination plus support for self-management**		
Fisher 2020	SEMCD	–0.13 (–1.06 to 0.81); p = 0.79
Fortin 2021	heiQ (Positive and Active engagement in life)	−0.04 (−0.13 to 0.05); p = 0.42
	heiQ (Health-directed behaviour)	0.05 (−0.12 to 0.22); p = 0.55
	heiQ (Skill and technique acquisition)	0.08 (−0.03 to 0.17); p = 0.17

	heiQ (Constructive attitudes and approaches)	−0.03 (−0.12 to 0.07); p = 0.57
	heiQ (Self-monitoring and insight)	0.23 (0.13 to 0.32); p < 0.01
	heiQ (Health service navigation)	
	heiQ (Social integration and support)	0.02 (−0.08 to 0.13); p = 0.64
	heiQ (Emotional wellbeing)	−0.02 (−0.16 to 0.13); p = 0.81
	SEMCD	0.08 (−0.21 to 0.37); p = 0.58
	Psychological distress (K6)	OR: 1.08 (0.57 to 2.03); p = 0.82
	High-risk alcohol consumption	OR: 1.24 (0.24 to 6.54); p = 0.80
	Smoking habit	OR: 2.40 (0.46 to 12.37); p = 0.30
	Physical activity	OR: 2.60 (1.37 to 4.93); p = 0.003
	Healthy eating	OR: 2.42 (1.30 to 4.50); p = 0.006
Stewart 2021	SEMCD	β-coefficient, (95% CI), –0.18 (–0.83 to 0.46); p = 0.58
	heiQ (Positive, active and engaged life)	β-coefficient, –0.07 (–0.23 to 0.10); p = 0.43
	heiQ (Health-directed behaviour)	β-coefficient, 0.05 (–0.15 to 0.25); p = 0.63
	heiQ (Skill and technique acquisition)	β-coefficient, –0.08 (–0.22 to 0.06); p = 0.24
	heiQ (Constructive attitudes and approaches)	β-coefficient, –0.11 (–0.26 to 0.05); p = 0.17
	heiQ (Self-monitoring and insight)	β-coefficient, 0.02 (–0.09 to 0.14); p = 0.68
	heiQ (Health service navigation)	β-coefficient, –0.06 (–0.21 to 0.08); p = 0.38
	heiQ (Social integration and support)	β-coefficient, –0.15 (–0.31 to 0.01); p = 0.06
	heiQ (Emotional wellbeing)	β-coefficient, –0.02 (–0.19 to 0.15); p = 0.84
	Psychological distress	OR: 1.49 (0.24 to 9.20); p = 0.67
	Health behaviours (No alcohol)	OR: 1.16 (0.14 to 9.45); p = 0.89
	Health behaviours (Physical activity ⩾2 times per week)	OR: 2.20 (0.60 to 8.11); p = 0.24
	Health behaviours (Good–excellent healthy eating)	OR: 1.79 (0.36 to 8.88); p = 0.48
	Health behaviours (Healthy BMI)	OR: 0.48 (0.07 to 3.43); p = 0.46
Mazya 2019	Mortality rate, percentage	18% vs 26%
Hong 2021	Average number of medications (Quality Improvement Programme vs Historical control)	5.80 vs 8.83; p < 0.001
	Average number of medications (Quality Improvement Programme vs Concurrent control)	5.80 vs 8.35; p = 0.001
Contant 2019	heiQ (Positive, active and engaged life)	OR: 1.72 (0.91 to 3.25); p = 0.09
	heiQ (Health-directed behaviour)	OR: 2.03 (1.16 to 3.55); p = 0.01
	heiQ (Skill and technique acquisition)	OR: 1.96 (1.13 to 3.39); p = 0.02
	heiQ (Constructive attitudes and approaches)	OR: 2.91 (1.45 to 5.84); p = 0.00
	heiQ (Self-monitoring and insight)	OR: 2.35 (1.02 to 5.40); p = 0.04
	heiQ (Health service navigation)	OR: 2.52 (1.21 to 5.21); p = 0.01
	heiQ (Social integration and support)	OR: 1.14 (0.58 to 2.24); p = 0.70
	heiQ (Emotional wellbeing)	OR: 1.97 (1.05 to 3.68); p = 0.04

Hochhalter 2010	Total Unhealthy Days	Coefficient: 0.07 (–0.64 to 0.78); p = 0.85
Salisbury 2018	Mean eight-item Morisky Medication Adherence Score	β-coefficient, 0.06 (–0.05 to 0.17); p = 0.27

MD: mean difference; CI: confidence interval; SD: standard deviation; SE: standard errors; OR: odds ratio; RR: relative risk; MARS-5: Medication Adherence Report Scale; SEAMS: Self-Efficacy for Appropriate Medication Use Scale; PIM: potentially inappropriate medication; MAI: Medication Appropriateness Index; DRP: drug-related problems; MRCI: Medication Regimen Complexity; heiQ: Health Education Impact Questionnaire; FAI: Frenchay Activities Index; NEADL: Nottingham Extended Activities of Daily Living; SEMCD = Stanford Chronic Disease Self-Efficacy 6-item Scale; SES6G = health related self-efficacy scale; MARS = Medication Adherence Report Scale; GSES = General Self-Efficacy Scale; K6 = Kessler 6-item Psychological Distress Scale Questionnaire; BMI: body mass index; IRR: incidence rate ratio; PIP: potentially inappropriate prescribing

Tab. 4: Results for sensitivity analysis

Outcomes	Treatment effects (95% CI)	No. of participants (studies)	Total heterogeneity, I^2 (%)
**Medicine Management Interventions**			
**Removal of High Risk of Bias Studies**			
**HRQoL**	**MD unless stated otherwise**		
Utility	0.028 higher (0.014 lower to 0.069 higher)	665 (2 RCT)	0
**Healthcare Utilisation**	**MD unless stated otherwise**		
Hospitalisation	0.13 more (0.23 fewer to 0.50 more)	53 (1 RCT)	-
Length of Hospital Stay	11.36 more (13.80 fewer to 36.52 more)	412 (2 RCT)	98
Emergency Department Visits	0.26 more (0.12 fewer to 0.65 more)	359 (1 RCT)	-
Outpatient Visits	0.46 more (1.26 fewer to 2.19 more)	359 (1 RCT)	-
**Support for Self-management Interventions**			
**Removal of High Risk of Bias Studies**			
**HRQoL**	**MD unless stated otherwise**		
Physical Component Score	**4.420 higher* (0.445 higher to 8.395 higher)**	50 (1 RCT)	-
Mental Component Score	0.690 lower (5.735 lower to 4.355 higher)	50 (1 RCT)	-
**Care Coordination plus Support for Self-management Interventions**			
**Removal of High Risk of Bias Studies**			
**HRQoL**	**MD unless stated otherwise**		
Physical Component Score	MD 0.802 higher (0.877 lower to 2.482 higher)	753 (5 RCT)	32
Mental Component Score	MD 0.662 higher (0.852 lower to 2.176 higher)	753 (5 RCT)	0
Other Quality of Life	SMD 0.108 higher (0.094 lower to 0.311 higher)	375 (2 RCT)	0
**Removal of NRSI**			
**Healthcare Utilisation**	**MD unless stated otherwise**		
Hospitalisation	0.01 fewer (0.07 fewer to 0.04 more)	3,354 (6 RCT)	0
Emergency Department Visits	0.02 more (0.15 fewer to 0.20 more)	1,751 (6 RCT)	0
General Practitioner Visits	3.26 more (0.64 fewer to 7.16 more)	3,370 (5 RCT)	100
Nurse Visits	0.34 fewer (4.26 fewer to 3.58 more)	1,971 (2 RCT)	96

CI: confidence interval; RCT: randomised control trial; NRSI: non-randomised studies of interventions; MD: mean difference; SMD: standardised mean difference; HRQoL: health-related quality of life; *: statistically significant
